# Remodeling Collagen Microenvironment in Liver Using a Biomimetic Nano‐Regulator for Reversal of Liver Fibrosis

**DOI:** 10.1002/advs.202300127

**Published:** 2023-04-23

**Authors:** Yan Liang, Jinjin Wang, Chenlu Xu, Wenshuai Han, Sixuan Wu, Yonghua Wu, Jingge Zhang, Junjie Liu, Zhenzhong Zhang, Jinjin Shi, Kaixiang Zhang

**Affiliations:** ^1^ School of Pharmaceutical Sciences Key Laboratory of Targeting Therapy and Diagnosis for Critical Diseases Collaborative Innovation Center of New Drug Research and Safety Evaluation Zhengzhou University Zhengzhou 450001 P. R. China

**Keywords:** collagen microenvironment, hepatic stellate cells, liver fibrosis, metalloproteinase‐1 mRNA silencing, platelet membranes, Zn (II) interference

## Abstract

Liver fibrosis is a progressive histological manifestation that happens in almost all chronic liver diseases. An unabated liver fibrosis may eventually develop into liver cirrhosis or hepatocellular carcinoma. Yet, the strategy for reversal of liver fibrosis is still limited. Herein, a biomimetic nano‐regulator (P‐ZIF8‐cirDNAzyme) is developed to affect both collagen synthesis and degradation in liver to remodel collagen microenvironment. It is found that Zn (II) interference can efficiently inhibit collagen synthesis in activated hepatic stellate cells (aHSC) by inactivating proline 4 hydroxylase and affecting many fibrosis‐related signaling pathways. Meanwhile, Zn (II)‐dependent circular DNAzymes (cirDNAzymes) are used to efficiently silence tissue inhibitors of metalloproteinase‐1, accelerating the degradation of collagen. They act in concert to recover the balance between collagen deposition and degradation. Additionally, ZIF‐8‐cirDNAzyme is coated by platelet membrane (PM) for precisely targeting aHSC via PM's inflammatory tropism and CD62p–CD44 interaction. In carbon tetrachloride‐induced fibrotic mice, P‐ZIF‐8‐cirDNAzyme shows a potent anti‐fibrotic effect, greatly reducing the expression of collagen by 73.12% and restoring liver function nearly to normal. This work proposes a prospective platform enabling ion interference and gene silencing, collectively acting in aHSC for reversal of liver fibrosis.

## Graphical abstract

The biomimetic nano‐regulator precisely targets hepatic stellate cell and produces Zn (II) interference and gene silencing. Zn (II) interference effectively inhibits collagen deposition simultaneously activating circular DNAzyme to silence tissue inhibitor of metalloproteinase 1 mRNA, which accelerates the degradation of accumulated collagen. This mutual promotion strategy recovers the balance between collagen deposition and degradation and remodels the collagen microenvironment.

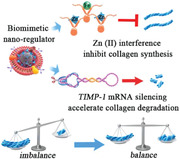

## 1 Introduction

Liver fibrosis is a progressive histological manifestation of chronic liver disease associated with alcohol abuse, viral infection, cholestasis, and autoimmune diseases.^[^ ^1^ ^]^ If not treated timely, liver fibrosis may eventually develop into liver cirrhosis and even hepatocellular carcinoma with high morbidity and mortality.^[^ ^2^ ^]^ Although several antifibrotic agents, including Resveratrol (for reduction of oxidative stress),^[^ ^3^ ^]^ obeticholic acid (for downregulation of collagen),^[^ ^4^ ^]^ and simtuzumab (for inhibition of collagen‐crosslinking),^[^ ^5^ ^]^ have been tested in clinical trials; limited efficacy and serious side effects remain major barriers limiting their application. Therefore, the development of efficient and specific strategy for reversal of liver fibrosis is highly desired.

Liver fibrosis is characterized by persistent activation of hepatic stellate cells (aHSC), leading to the imbalance between collagen deposition and degradation.^[^ ^6^ ^]^ Specifically, aHSC contain Proline 4 hydroxylase (P4H) enzyme with high activity. P4H can promote the formation of stable collagen by catalyzing the hydroxylation of collagen proline residues,^[^ ^7^ ^]^ which is a major mechanism for inducing large amount of collagen deposition.^[^ ^8^ ^]^ In addition, the tissue inhibitor of metalloproteinase 1 (TIMP‐1) in aHSC is overexpressed, which can bind and inhibit matrix metalloproteinase (MMP) in liver. Since MMP is an essential enzyme in degradation of collagen, the highly expressed TIMP‐1 is considered as another central mechanism for inducing liver fibrosis.^[^ ^9^ ^]^ Therefore, simultaneously regulating P4H and TIMP‐1 in aHSC may be a potential strategy to disturb the imbalance between collagen deposition and degradation for reversal of liver fibrosis.

Since P4H enzyme is a member of Fe (II)‐dependent dioxygenase superfamily,^[^ ^10^ ^]^ ion interference could be an attractive strategy for inhibiting P4H enzyme activity. It has been reported that Zn (II) can efficiently inhibit P4H activity by replacing the Fe (II) core in the enzyme.^[^ ^11^ ^]^ Interestingly, lots of clinical investigations have shown that serum zinc deficiency is common in patients with chronic liver disease.^[^ ^12^ ^]^ It has also been demonstrated that Zn (II) has great potential in liver protection.^[^ ^13^ ^]^ However, the use of “Zn (II) interference” in aHSC for inhibiting P4H enzyme activity has not been reported.

Except for inhibiting P4H enzyme activity, to recover balance between collagen deposition and degradation, strategies for promoting the degradation of deposited collagen are needed. Gene therapy has been applied as an efficient strategy for downregulation of collagen level in liver by using siRNA to interfere TIMP‐1 mRNA expression.^[^ ^14^ ^]^ However, the gene silencing efficacy remains limited due to the relatively low stability of siRNA. DNAzyme is a single‐stranded DNA molecule with excellent RNA cleavage activity in the presence of metal ion.^[^ ^15^ ^]^ Compared with siRNA, DNAzyme possesses attractive biostability and does not perform through the formation of RNA‐induced silencing complex.^[^ ^16^ ^]^ But precisely delivering DNAzyme to aHSC and providing enough metal cofactor for implementing the RNA‐cleaving activity remain a challenge.

Nano‐scaled zeolitic imidazolate framework‐8 (ZIF‐8) nanoparticles have been developed as a nanocarrier for DNAzyme delivery. Benefiting from its pH‐responsive structural collapse, ZIF‐8 can escape from lysosome and concurrently release Zn (II) and DNAzyme into cytoplasm,^[^ ^17^ ^]^ becoming a smart self‐driven nanoplatform and allowing for simultaneous ion interference and gene silencing to regulate aHSC. Meanwhile, it is critical to deliver nano‐formulation into the effector cells (aHSC) to exert maximum therapeutic effect. PM exhibits natural aggregation and adhesion to inflammatory sites.^[^ ^18^ ^]^ More importantly, its surface protein CD62p can specifically bind to the CD44 receptor which is highly expressed by aHSC.^[^ ^19^ ^]^ Meanwhile, PM has an excellent ability to escape elimination by mononuclear macrophage system and avoid inducing apparent immune response, showing great potential for targeted delivery of nano‐formulation to aHSC.

**Scheme 1:**
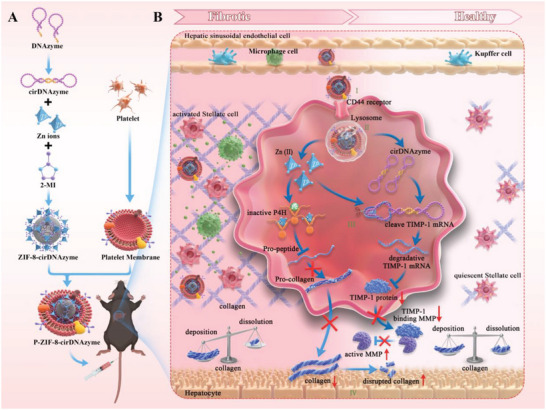
The scheme of aHSC‐targeted biomimetic nano‐regulator based on P‐ZIF8‐cirDNAzyme A) Schematic diagram of the procedure for preparing P‐ZIF‐8‐cirDNAzyme. B) The mechanism of P‐ZIF‐8‐cirDNAzyme precisely and effectively remodeling the collagen microenvironment and reversal of liver fibrosis.

Herein, we designed P‐ZIF‐8‐cirDNAzyme, an aHSC‐targeted biomimetic nano‐regulator composed of platelet membrane, ZIF‐8, and TIMP‐1 circular DNAzyme (cirDNAzyme), which acts on the dual pathway of inhibiting collagen synthesis and promoting collagen degradation to remodel the collagen microenvironment. As shown in **Scheme** **1**, I) with the help of platelet membrane, the nano‐regulator successfully escaped from the phagocytosis of mononuclear macrophage and Kupffer cells, and precisely targeted aHSC via CD62p‐CD44 mediated specific binding; II) due to the proton sponge effect of ZIF‐8, nano‐regulator escaped lysosomes and released Zn (II) and cirDNAzyme; III) released Zn (II) effectively inhibited collagen deposition, while simultaneously activating cirDNAzyme to silence TIMP‐1 mRNA, which accelerated the degradation of accumulated collagen; and IV) this mutual promotion strategy recovered the balance between collagen deposition and degradation in liver and remodeled the collagen microenvironment. This rational designed nanodrug offers an attractive therapeutic platform for specifically and efficiently remodeling the collagen microenvironment and delaying the progression of liver disease.

## 2 Results and Discussion

### 2.1 Synthesis and Characterization of Biomimetic Nano‐Regulator (P‐ZIF‐8‐cirDNAzyme)

**Figure 1:**
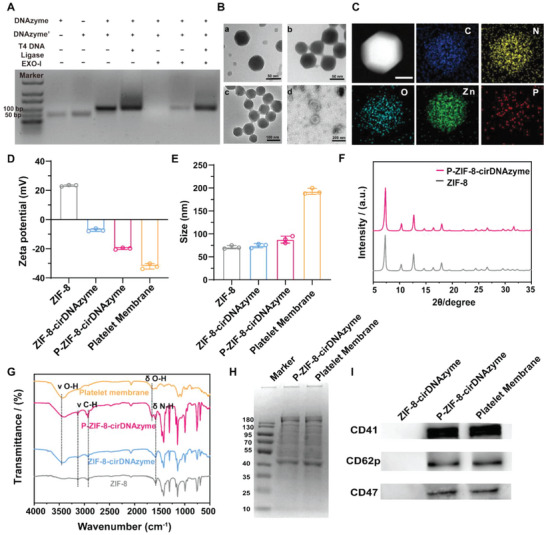
Synthesis and characterization of P‐ZIF‐8‐cirDNAzyme. A) cirDNAzyme's self‐assembly process and the stability with Exonuclease I (Exo‐) treatment. B) Representative TEM image of a) ZIF‐8, b) ZIF‐8‐cirDNAzyme, c) P‐ZIF‐8‐cirDNAzyme, and d) platelet membrane. C) High‐angle annular dark‐field (HAADF) images of P‐ZIF‐8‐cirDNAzyme and corresponding element mapping images of C, N, O, Zn, and P; Scale bar: 100 nm. D,E) Zeta potential (D) and size (E) of ZIF‐8, ZIF‐8‐cirDNAzyme, P‐ZIF‐8‐cirDNAzyme, and platelet membrane (*n* = 3). The data are presented as the mean ± SD from three independent experiments. F) X‐ray diffraction (XRD) patterns of ZIF‐8 and P‐ZIF‐8‐cirDNAzyme. G) Fourier Transform Infrared spectroscopy (FT‐IR) of ZIF‐8, ZIF‐8‐cirDNAzyme, P‐ZIF‐8‐cirDNAzyme, and platelet membrane. H) Sodium dodecyl sulfate‐polyacrylamide gel electrophoresis (SDS‐PAGE) of P‐ZIF‐8‐cirDNAzyme and platelet membrane. I) Western blot of ZIF‐8‐cirDNAzyme, P‐ZIF‐8‐cirDNAzyme, and platelet membrane.

To synthesis the biomimetic nano‐regulator (P‐ZIF‐8‐cirDNAzyme), we first needed to design a TIMP‐1 DNAzyme. The “8‐17” deoxyribozyme was selected and converted into a therapeutic DNAzyme that specifically recognizes and cleaves TIMP‐1 mRNA. It has been reported that cyclization of the DNA structure could further improve the stability of oligonucleotide drugs in vivo,^[^ ^20^ ^]^ so we constructed a cirDNAzyme to achieve high mRNA‐silencing efficiency. CirDNAzyme was synthesized through the hybridization of complementary TIMP‐1 DNAzyme sequences, and T4 DNA ligase was used to achieve a highly efficient nick closing. The successful preparations of cirDNAzyme were verified by agarose gel electrophoresis, and we confirmed that cirDNAzyme can resist degradation by exonuclease I (widely found in biological media) (**Figure** **1**A). Then we prepared ZIF‐8‐cirDNAzyme nanoparticles through a simple one‐step synthesis procedure. Specifically, cirDNAzymes were first premixed with a solution containing zinc ions, followed by addition of 2‐methylimidazole, initiating ZIF‐8‐DNAzyme self‐assembly and bringing about turbidity in the solution. After that, purified platelet membranes were mixed and sonicated with the ZIF‐8‐cirDNAzyme to obtain the final nanoparticles (P‐ZIF‐8‐cirDNAzyme).

To characterize the successful preparation of nanoparticles, transmission electron microscopy (TEM) was first performed. It showed that P‐ZIF‐8‐cirDNAzyme displayed a uniform spherical structure and with a core–shell morphology (Figure 1B). Besides, the corresponding element mapping images indicated that the P‐ZIF‐8‐cirDNAzyme contained the characteristic zinc element from ZIF‐8 and the phosphorus element from cirDNAzyme (Figure 1C). As shown in Figure 1D, the loading of cirDNAzyme significantly reduced the surface zeta potential of ZIF‐8, and the surface zeta potential further decreased after platelet membrane modification. Moreover, ZIF‐8‐cirDNAzyme nanoparticle has a larger diameter than ZIF‐8 nanoparticles, and platelet membrane coating further increases the size, resulting in an average diameter of P‐ZIF‐8‐cirDNAzyme ≈88 nm (Figure 1E). Both the surface zeta potential and size change suggested successful loading of DNAzymes and coating of PM in preparation, which was further confirmed by thermogravimetric analysis (TGA) and Ultra Violet scanning (UV‐Vis) (Figure S1and S2, Supporting Information). To test the loading of cirDNAzymes, the loading efficiency was quantified by agarose gel electrophoresis (Figure S3, Supporting Information), a high rate of cirDNAzyme incorporation was demonstrated (3.57 wt% with the input amount of 90 µg mL^−1^), likely due to the strong electrostatic interaction between the metal nodes of the framework and cirDNAzyme's backbone phosphates, as well as the porous structure confinement of the nanoparticles.^[^ ^21^ ^]^ Additionally, we tested that the loading of Zn(II) in P‐ZIF‐8‐DNAzyme was about 19.54 wt% via inductively coupled plasma mass spectrometry (ICP‐MS). This provided a foundation for regulation of intracellular zinc ion concentration.

Afterward, X‐ray diffraction (XRD) analysis revealed the P‐ZIF‐8‐cirDNAzyme retained a high crystallinity (Figure 1F), and Fourier transform infrared spectroscopy (FT‐IR) was performed on different nanoparticles. As shown in Figure 1G, the peaks at 3119 and 2945 cm^−1^ correspond to the stretching vibrations of C—H in imidazole rings of ZIF‐8 and the peak at 3440 cm^−1^ correspond to the stretching vibration of O—H in organic materials (here may be mainly cirDNAzyme). And only the preparation containing PM showed a peak at 1653 cm^−1^, indicating that the flexural vibration of O—H in PM is presented. All the evidence above supported the successful fabrication of P‐ZIF‐8‐cirDNAzyme.

To confirm the successful translocation of PM onto the surface of P‐ZIF‐8‐cirDNAzyme, which can protect nanoparticles from immune clearance, we analyzed the protein composition of P‐ZIF‐8‐cirDNAzyme by sodium dodecyl sulfate‐polyacrylamide gel electrophoresis. It was found that the protein profiles were very similar between P‐ZIF‐8‐cirDNAzyme and PM (Figure 1H). Furthermore, Western blot further showed that P‐ZIF‐8‐cirDNAzyme had the characteristic protein of platelet membrane, including CD47, CD41, and CD62p (Figure 1I), which provided the basis for escaping the phagocytosis of macrophage and targeting aHSC in vivo. Importantly, prothrombotic and platelet‐activating molecules such as thrombin, adenosine diphosphate (ADP), and thrombin B2 (TXB2) were removed from the nanoparticles, preventing the P‐ZIF‐8‐cirDNAzyme from developing a thrombotic response, which ensured the possibility of further application in vivo (Figure S4, Supporting Information).

### 2.2 In Vitro Test of P‐ZIF‐8‐cirDNAzyme for Inhibition of P4H Activity and Silencing TIMP‐1 mRNA Expression

**Figure 2:**
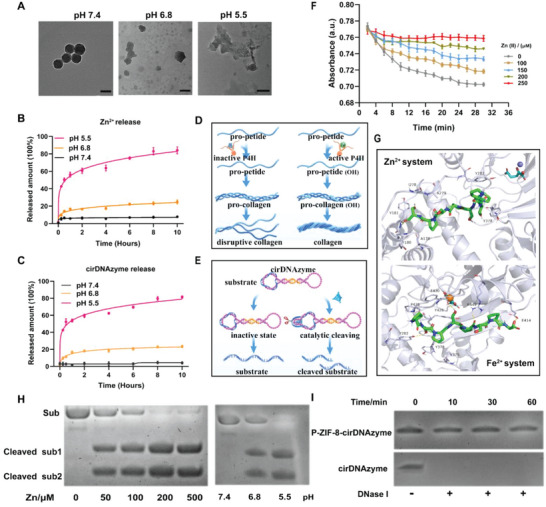
In vitro functional properties of P‐ZIF‐8‐cirDNAzyme. A) TEM images of the degradation of P‐ZIF‐8‐cirDNAzyme at pH 7.4/6.8/5.5. Scale bar: 100 nm. B,C) In vitro Zn (II) (B) and cirDNAzyme (C) release curves of P‐ZIF‐8‐cirDNAzyme at pH 7.4/6.8/5.5 (*n* = 3). The data are presented as the mean ± SD. D) The schematic diagram of Zn (II) mediated inhibition of P4H activity and prevent collagen deposition. E) The schematic diagram of Zn (II) activated cirDNAzyme shearing the TIMP‐1 mRNA substrate. F) P4H activity assay kit analyzes the catalyzed activity of P4H at different concentrations of Zn (II) (*n* = 3). The data are presented as the mean ± SD. G) Molecular dynamic simulation characterizes the binding mode of P4H and substrate peptide at Fe (II) and Zn (II) systems. The purple ball and the brown ball represent the Zn and Fe ions, respectively. Gray cartoon represents P4H enzyme, colorful sticks represent substrate peptide. The combinations of numbers and letters (A279 and Y282) represent amino acid serial number. H) CirDNAzymes mediated cleavage reaction under different concentrations of Zn (II) and P‐ZIF‐8‐cirDNAzyme mediated cleavage efficiency at different pH 7.4/6.8/5.5. I) PAGE analysis of the degradation of cirDNAzyme and P‐ZIF‐8‐cirDNAzyme exposed to DNase I. Results are presented as means ± SD from three independent experiments.

It has been reported that the Zn—N bond in ZIF‐8 could respond to low pH for dissociation which is a widely used mechanism for pH‐sensitive drug release.^[^ ^22^ ^]^ Combined with the unstable feature of PM under acidic conditions,^[^ ^23^ ^]^ it is expected that the P‐ZIF‐8‐cirDNAzyme can release Zn (II) and cirDNAzymes in low pH environment. To investigate the drug release behavior of the nanoplatform, P‐ZIF‐8‐cirDNAzyme was incubated at different pH solution. TEM showed that the morphology of P‐ZIF‐8‐cirDNAzyme collapsed in acidic pH solution, indicating the acidic sensitivity of ZIF‐8 (**Figure** **2**A). The conclusion was also verified by the UV–vis spectrum absorbance (Figure S5, Supporting Information). Next, we quantitatively analyzed Zn (II) and cirDNAzymes released from nanoparticles (Figure 2B,C). At pH 5.5, the final release rates of Zn (II) and cirDNAzyme reached 84.1% and 80.6%, respectively, after 10 h incubation, while the final release rates of Zn (II) and cirDNAzymes were only 7.76% and 4.11%, respectively, at pH 7.4, which ensured very low extracellular leakage and efficient intracellular release, laying the foundation for subsequent intracellular biological functions of Zn (II) and cirDNAzymes (Figure 2D,E).

To detect the effect of Zn (II) on P4H enzyme, we measured the activity of P4H under different Zn (II) concentration with an activity detection kit. It was found that Zn (II) inhibited P4H enzymatic reaction in a dose‐dependent manner (Figure 2F). To further determine the molecular behavior of Zn (II) for inhibition of P4H, we used molecular dynamic simulation to explore the difference in the interaction between the P4H and its substrate polypeptide in Zn (II) and Fe (II) systems. As shown in Figure 2G, in Fe (II) systems, P4H maintained a stable conformation with the substrate polypeptide through five hydrogen bonding and hydrophobic interaction with amino acids (Pro482, Phe430, Val376, and Ile365). By contrast, in Zn (II) system, only one hydrogen bond was formed, and hydrophobic interactions were with Ala279, Ile278, and Ala178. These alterations in the binding mode between P4H and the substrate polypeptide might affect their binding forces. Thus, the molecular mechanics/generalized Born surface area (MM/GBSA) method was used to calculate the bound free energy of P4H and the substrate polypeptide under different metal ion systems. As expected, Zn (II) could significantly reduce the binding ability between the substrate polypeptide and P4H by nearly1.3 fold (Table S1, Supporting Information).

Further analysis was then performed to determine whether P‐ZIF‐8‐cirDNAzyme can release adequate Zn (II) serving as cirDNAzyme's cofactor for biocatalysis. First, we verified the function of cirDNAzyme to silence TIMP‐1 substrate. As expected, the substrate‐cleaving efficiency displayed tight Zn (II) concentration‐dependence by polyacrylamide gel electrophoresis (PAGE) analysis (Figure 2H). Next, we investigated the TIMP‐1 mRNA‐cleavage activity of P‐ZIF‐8‐cirDNAzyme. As shown in Figure 2H, it was proved that P‐ZIF‐8‐cirDNAzyme could completely cleave the substrate under pH 5.5, while the cleavage activity was negligible at pH 7.4. These results demonstrated that P‐ZIF‐8‐cirDNAzyme could provide adequate Zn (II) and cirDNAzyme for effective TIMP‐1 silencing.

To explore the stability of P‐ZIF8‐cirDNAzyme, we dispersed PM‐coating and non‐PM‐coating nanoparticles in phosphate‐buffered saline (PBS) and 10% fetal bovine serum (FBS) over the course of 1 week. There were no obvious changes in the size and polydispersity index (PDI) of P‐ZIF‐8‐cirDNAzyme during 1 week. Besides, the encapsulation efficiency of cirDNAzyme in P‐ZIF‐8‐DNAzyme is still nearly 95% in seventh day. As a comparison, the size and PDI of ZIF‐8‐cirDNAzyme gradually increased, and the encapsulation efficiency was getting lower during the week, indicating that nanoparticle stabilization can be facilitated by cell membrane coating (Figure S6, Supporting Information). To confirm nano‐encapsulation maintains cirDNAzyme integrity, we evaluated the stability of cirDNAzyme in DNase I solution, we found that even exposed P‐ZIF‐8‐cirDNAzyme to DNase I for 60 min, no obvious cirDNAzyme degradation was observed (Figure 2I). It demonstrated that the packing of ZIF‐8 and the coating of PM greatly improved cirDNAzymes’ stability, which is essential for intracellular application.

### 2.3 Intracellular Zn (II) Interference and TIMP‐1 Silence Ability of P‐ZIF‐8‐cirDNAzyme

**Figure 3:**
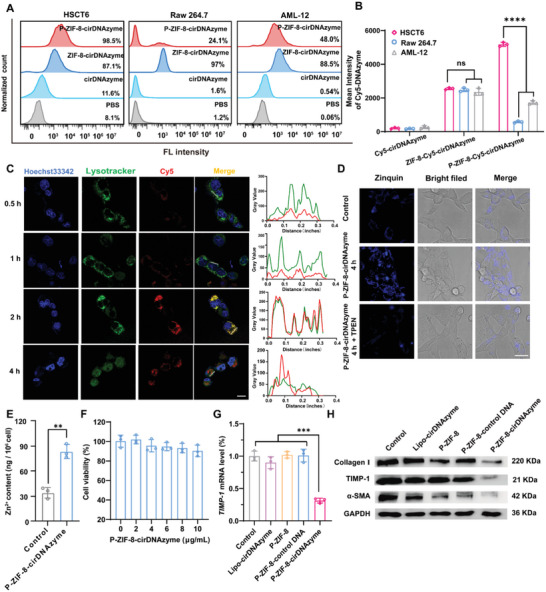
P‐ZIF‐8‐cirDNAzyme uptake, distribution, and function in cells. A,B) Flow cytometry analysis (A) and semi‐quantitative data (B) in HSCT6, Raw 264.7, and AML‐12 cells incubated with Cy5‐cirDNAzyme, ZIF‐8‐Cy5‐cirDNAzyme, and P‐ZIF‐8‐Cy5‐cirDNAzyme, respectively (*n* = 3). C) Fluorescent imaging of the subcellular localization of P‐ZIF‐8‐cirDNAzyme in HSCT6 cells after different incubation times. Scale bar: 15 µm. The spatial co‐localization of cirDNAzyme and lysosomes were statically analyzed by Image J. D) CLSM images of intracellular free Zn (II) production after treatment with P‐ZIF‐8‐cirDNAzyme in HSCT6. Scale bar: 35 µm. E) ICP‐MS analysis of Zn (II) levels in HSCT6 after P‐ZIF‐8‐cirDNAzyme treatment (*n* = 3). F) Cell viability of HSCT6 cells subjected to different concentrations of P‐ZIF‐8‐cirDNAzyme (*n* = 3). G) RT‐qPCR analysis of TIMP‐1 mRNA in HSCT6 after treatment with P‐ZIF‐8‐cirDNAzyme (*n* = 3). H) Western blot analysis of Collagen I, TIMP‐1, and *α*‐SMA protein in HSCT6 with different treatments. All data are presented as the mean ± SD for E), statistical analysis was performed using two‐tailed unpaired Student's *t*‐tests (**P < 0.01). For (B) and (G), statistical analysis was calculated via one‐way ANOVA with Tukey's post‐test (***P < 0.001 and ****P < 0.0001; ns: no significance).

Next, we investigated how P‐ZIF‐8‐cirDNAzyme interacted with different cells, including aHSC (HSCT6), macrophages (Raw 264.7) and mouse hepatocyte (AML‐12). To conduct the study, we labeled cirDNAzyme with Cy5 and investigated the uptake differences of nanoparticles among HSCT6, Raw 264.7, and AML‐12. The flow cytometry analysis (**Figure** **3**A) showed that, compared with 24.1% uptake efficiency for Raw 264.7 and 48.0% for AML‐12, P‐ZIF‐8‐cirDNAzyme was more efficiently internalized into HSCT6 cells with 98.5% uptake efficiency. The confocal laser scanning microscopy (CLSM) images showed a similar phenomenon (Figure S7, Supporting Information). Next, we studied the interaction mechanism between P‐ZIF‐8‐cirDNAzyme and HSCT6. As shown in Figure S8, Supporting Information, compared with P‐ZIF‐8‐cirDNAzyme treated group, the fluorescence intensity of Cy5 obviously decreased with anti‐CD62p or anti‐CD44 antibody treated, suggesting that CD62p‐CD44‐mediated actively targeting mechanism facilitates the uptake of P‐ZIF‐8‐cirDNAzyme into HSCT6 cells, which has also been reported in previous ^[^ ^24^ ^]^. We also measured CD44 expression in different cells. A higher expression level of CD44 protein was found on HSCT6 cells than that on AML‐12 cells and Quiescent stellate cells (Figure S9, Supporting Information), which guaranteed the excellent internalization capability of P‐ZIF‐8‐cirDNAzyme. These results suggested that platelet membrane encapsulation significantly reduced the clearance of the nanoparticles by macrophages (Raw 264.7), nonspecific uptake of hepatocytes (AML‐12), and promoted the uptake of aHSC (HSCT6).

After efficient cell internalization, to achieve cytoplasmic gene silencing, the escape of cirDNAzymes from lysosomes is essential. To visualize the endosomal escape process, cirDNAzymes were labeled with Cy5 and the endosomes were stained with lysotracker green. As shown in Figure 3C, P‐ZIF‐8‐cirDNAzyme localized to lysosomes at 2 h after incubation, which was confirmed by overlapping fluorescence between cirDNAzyme and lysosome. In contrast, 4 h after transfection, efficient dissociation between cirDNAzyme and lysosome was observed, indicating efficient release of cirDNAzyme into the cytoplasm. This is caused by the dissociation of the ZIF‐8 scaffold, more specifically, the protonation of the imidazole ring in MOF in the acidic pH of the endosomes.^[^ ^25^ ^]^

Subsequently, to evaluate the intracellular free Zn (II) level, we used Zinquin AM, a Zn (II) probe, to detect the alteration of intracellular Zn (II) concentration after incubated with P‐ZIF‐8‐cirDNAzyme. We found that the blue fluorescent intensity obviously increased after P‐ZIF‐8‐cirDNAzyme treatment (Figure 3D). Afterward, TPEN, a Zn‐specific chelator treated group attenuated the blue fluorescence, which confirmed that the increased fluorescence intensity is caused by the accumulation of free Zn (II), and the ICP‐MS results further quantified the increase of intracellular Zn (II) content from 55.49 to 89.81 ng per 10^6^ cells (Figure 3E), which indicated an effective intracellular accumulation of free Zn (II) and laid the foundation for the subsequent inhibition of the activity of P4H enzyme and the activation of cirDNAzyme shearing activity. Since the biosafety of a delivery system is important for its future application, we tested the cytotoxicity of P‐ZIF‐8‐cirDNAzyme, CCK8 assayed that the P‐ZIF‐8‐cirDNAzyme did not induce obvious cell cytotoxicity at a concentration scope of 0–10 µg mL^−1^ (Figure 3F). We also tested the cytotoxicity of P‐ZIF‐8‐cirDNAzyme in hepatocytes, and there was no obvious cytotoxic observed (Figure S10, Supporting Information).

Next, we evaluated the potential of P‐ZIF‐8‐cirDNAzyme for TIMP‐1 silencing. As reverse transcription‐quantitative real time polymerase chain reaction (RT‐qPCR) assay showed, compared with the other four groups (Control, Lipo‐cirDNAzyme, P‐ZIF8, and P‐ZIF8‐control‐DNA), P‐ZIF‐8‐cirDNAzyme treatment could downregulate TIMP‐1 mRNA to 31.3% (Figure 3G), which reflected the excellent gene silencing function of cirDNAzyme. Afterward, we further tested synergistically gene silencing and Zn (II) interference produced effects on collagen. We found that both immunofluorescence (Figure S11, Supporting Information) and Western blot (Figure 3H) results showed that P‐ZIF‐8‐cirDNAzyme could significantly decrease collagen levels. Additionally, Western blot results showed that the TIMP‐1 and *α*‐smooth muscle actin (α‐SMA) protein in P‐ZIF‐8‐cirDNAzyme were also reduced (Figure 3H). These results demonstrated that P‐ZIF‐8‐cirDNAzyme provides a promising strategy for the breakdown of collagen and anti‐fibrosis.

### 2.4 RNA Sequencing Analysis of Anti‐Fibrosis Activity of P‐ZIF‐8‐cirDNAzyme in HSCT6

To further explore the anti‐fibrosis mechanisms of P‐ZIF‐8‐cirDNAzyme, RNA sequencing of HSCT6 cells after treatment with P‐ZIF‐8‐cirDNAzyme was implemented, and untreated HSCT6 cells were used as a control. Principal component analysis revealed that the sample data between control and P‐ZIF‐8‐cirDNAzyme treated groups were scattered, suggesting high‐quality of RNA sequencing (Figure S12A, Supporting Information). The result of the heat map showed the sample duplication within groups and the difference between groups. As expected, a high correlation (*R* ^2^ > 0.95) was in the same treatment groups, but no significant correlations between control and P‐ZIF‐8‐cirDNAzyme (Figure S12B, Supporting Information), further confirming that different genes were specifically caused by the treatment with P‐ZIF‐8‐cirDNAzyme.

**Figure 4:**
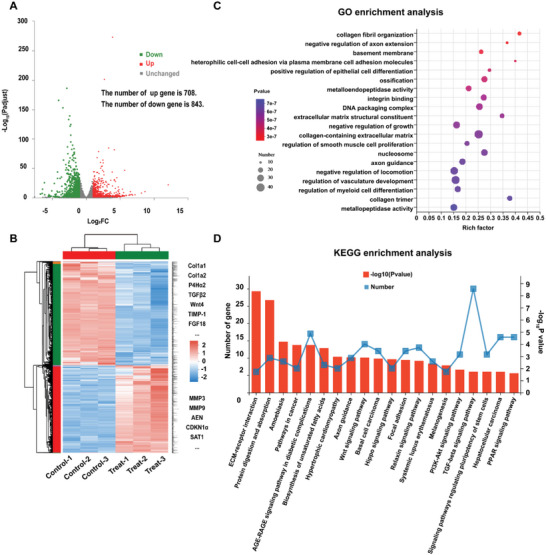
RNA sequencing analysis in HSCT6 cells subjected to P‐ZIF‐8‐cirDNAzyme treatment. A) Volcanic map of differentially expressed genes (DEGs). The up‐regulated genes are represented by red dots and the down‐regulated genes by green dots. B) Clustering heat map of DEGs. The abscissa is the sample name, and the ordinate is the normalized value of the DEGs. The red color shows the higher expression level, and the blue shows the lower expression level. C) Gene Ontology (GO) enrichment analysis. The vertical axis represents the GO term, and the horizontal axis represents Rich factor (the ratio of sample number to the background number in the GO term). The larger the Rich factor, the greater the degree of enrichment. The size of the dots indicates the number of genes/transcripts in this GO term, and the color of the dots corresponds to different *P* value ranges. D) Kyoto encyclopedia of genes and genomes (KEGG) enrichment analysis. The horizontal axis represents the KEGG pathway, and the *y*‐coordinate on the left represents the number of genes/transcripts in this pathway, corresponding to different points on the broken line. The ordinate on the right represents the enrichment significance level, corresponding to the column height. The larger −log10 (P value) represents more significantly in the KEGG pathway.

The result of the volcano map of differentially expressed genes (DEGs) further revealed that, of the 1551 DEGs, 843 (54%) were downregulated and 708 (46%) were upregulated in P‐ZIF‐8‐cirDNAzyme treated group (**Figure** **4**A). Subsequently, by clustering of these DEGs (Figure 4B), we found that in P‐ZIF‐8‐cirDNAzyme treated group, not only the TIMP‐1 gene was downregulated, but many genes related to collagen generation were also greatly downregulated, including Col1a1, Col2a2, and P4H*α*2, which indicated that our preparation had a significant effect on collagen collapse. To our surprise, with P‐ZIF‐8‐cirDNAzyme treatment, some genes that promote fibrosis (TGF*β*2 and Wnt4) were downregulated, while MMPs that promote collagen breakdown and several genes that promote apoptosis and inhibit proliferation were upregulated. Additionally, as illustrated by the gene ontology (GO) enrichment analysis (Figure 4C), the DEGs were mainly involved in pathways related to collagen fibril organization, extracellular matrix structural constituent, collagen‐containing extracellular matrix, and collagen trimer. These collagen‐related pathways were all influenced after P‐ZIF‐8‐cirDNAzyme treatment. Conformably, Kyoto encyclopedia of genes and genomes (KEGG) enrichment analysis (Figure 4D) showed that the DEGs were related to collagen signaling pathways (ECM–receptor interaction). More importantly, we found that DEGs in P‐ZIF‐8‐cirDNAzyme treated were enriched in multiple fibrosis‐related signaling pathways (Wnt, PI3K‐Akt, and TGF‐*β*), which broadly participate in the fibrosis process, such as activation and proliferation of hepatic stellate cells, the polarization of Kupffer cells, and ECM production. In short, these results above implied that P‐ZIF‐8‐cirDNAzyme not only inhibited P4H activity and silenced TIMP‐1 mRNA as demonstrated in our experiments but also could participate in multiple anti‐fibrotic pathways more broadly.

### 2.5 In Vivo Tissue Distribution and Safety of P‐ZIF‐8‐cirDNAzyme

Inspired by the excellent performance in vitro, the therapeutic effect of P‐ZIF‐8‐cirDNAzyme in vivo was further explored. First, we tested P‐ZIF‐8‐cirDNAzyme's biocompatibility in mice. P‐ZIF‐8‐cirDNAzyme consisted of excellent hemocompatibility under repeated intravenous dosing within 6 weeks (Figure S13, Supporting Information). Next, Hematoxylin and eosin (H&E) staining revealed that P‐ZIF‐8‐cirDNAzyme did not cause significant organ (e.g., heart, kidneys, liver, lung, and spleen) damage after repeated administration (Figure S14, Supporting Information). These results indicated that the P‐ZIF‐8‐cirDNAzyme had no significant toxicity during the 6 weeks of treatment.

**Figure 5:**
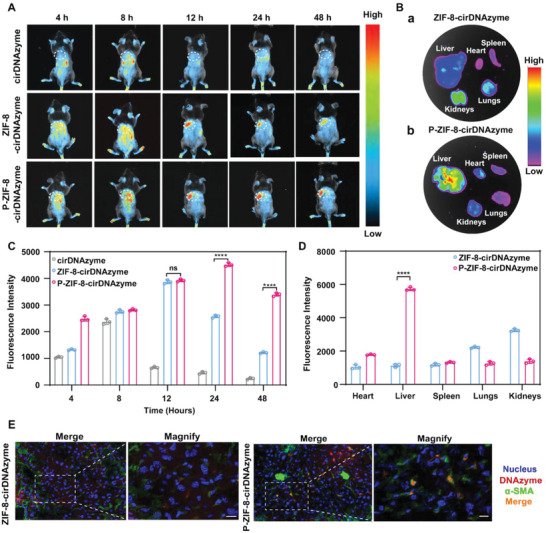
Tissue distribution and cellular localization of P‐ZIF‐8‐cirDNAzyme in mice with CCl_4_‐induced liver fibrosis. A) Representative in vivo fluorescent pictures of fibrotic mice treated with cirDNAzyme, ZIF‐8‐cirDNAzyme, and P‐ZIF‐8‐cirDNAzyme. The white dashed circle represents the liver site. B) Fluorescence images of liver tissues of fibrotic mice treated with ZIF‐8‐cirDNAzyme and P‐ZIF‐8‐cirDNAzyme. C,D) Semiquantitative analysis of fluorescence intensity of (A) and (B). All data are presented as the mean ± SD, *n* = 3. The statistical analysis was performed using two‐tailed unpaired Student's *t*‐tests (****P < 0.0001; ns: no significance). E) Representative scanning immunofluorescence images of liver uptake and cellular distribution of P‐ZIF‐8‐cirDNAzyme and ZIF‐8‐cirDNAzyme in fibrotic mice. Merge (10×), Magnify (90×) Scale bar: 20 µm.

Upon confirming P‐ZIF‐8‐cirDNAzyme safety in vivo, we next sought to evaluate the in vivo behavior of the nano‐formulation. First, to investigate the biodistribution of nanoparticles, two Cy5‐labeled cirDNAzyme nanoparticles (P‐ZIF‐8‐cirDNAzyme and ZIF‐8‐cirDNAzyme) were injected via the tail vein into the carbon tetrachloride (CCl_4_)‐induced fibrotic mice and naked Cy5‐cirDNAzyme was used as a control group. As shown in **Figure** **5**A, naked cirDNAzyme was mainly concentrated in the kidneys and quickly cleared after 8 h. Whereas, ZIF8‐cirDNAzyme and P‐ZIF8‐cirDNAzyme were largely accumulated in the liver and effectively extended the retention time, especially P‐ZIF8‐cirDNAzyme still retain a strong intensity even though at 48 h. Additionally, the results of isolated organ imaging also confirmed that the accumulation in the liver of the group P‐ZIF‐8‐cirDNAzyme was significantly 5.08‐fold stronger than that of group ZIF‐8‐cirDNAzyme (Figure 5B). The semi‐quantitative data of fluorescence intensity showed that despite the accumulation fluorescence value of liver between ZIF‐8‐cirDNAzyme and P‐ZIF‐8‐cirDNAzyme at 12 h was similar, the P‐ZIF‐8‐cirDNAzyme group was significantly higher than the ZIF‐8‐cirDNAzyme group at 24 h (Figure 5C,D). These findings were consistent with the literature that the platelet membrane tends to adhere and aggregate at the inflammation site of the liver. ^[^ ^18a^ ^]^ Notably, there are a large number of parenchymal cells in the liver, such as hepatocytes, which are mainly responsible for the metabolism of nanoparticles. Thus, we still needed to verify whether the nanoparticles could precisely target aHSC in the liver. Immunofluorescence images showed that the aHSC marker (*α*‐SMA, green) colocalized with the fluorescence signal of the nanoparticles (Cy5‐cirDNAzyme, red) in P‐ZIF‐8‐cirDNAzyme group (Figure 5E, right), indicating specific uptake of aHSC. The fluorescence signal of ZIF‐8‐cirDNAzyme nanoparticles was weaker than that of the P‐ZIF‐8‐cirDNAzyme group and did not overlap with the aHSC signal (Figure 5E, left). These results indicated that platelet membrane coating could greatly improve the accumulation of the preparation in the liver and selectively target aHSC.

### 2.6 Anti‐Fibrotic Activity of P‐ZIF‐8‐cirDNAzyme In Vivo

**Figure 6:**
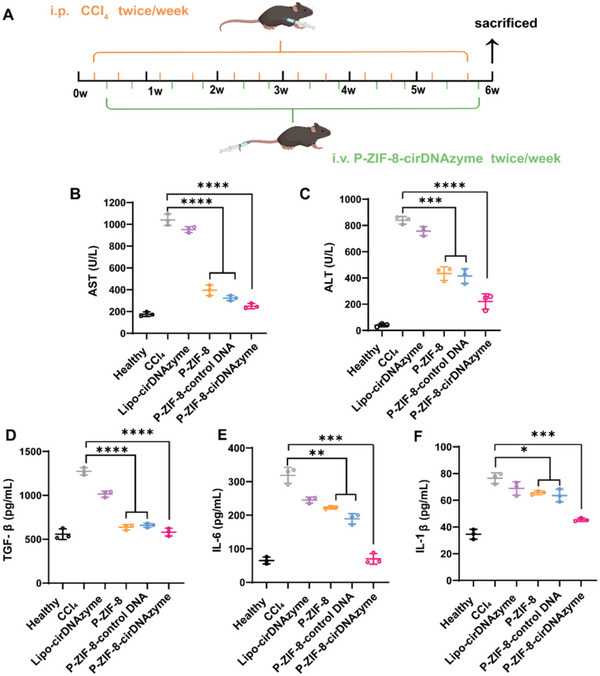
Serum detection of liver enzyme, proinflammatory cytokines in mice of CCl_4_‐induced liver fibrosis with different treatments. A) Schedule of the therapeutic process of P‐ZIF‐8‐cirDNAzyme. B,C) Liver enzyme activity tests of ALT and AST of mice with CCl_4_‐induced liver fibrosis with various treatments (*n* = 3). D–F) Cytokines in the serum of mice with CCl_4_‐induced liver fibrosis with various treatments (*n* = 3). All data are presented as the mean ± SD. The statistical significance was calculated via one‐way ANOVA with Tukey's post‐test (*P < 0.05, **P < 0.01, ***P < 0.001, and ****P < 0.0001; ns: no significance).

To evaluate the anti‐fibrosis effect of P‐ZIF‐8‐cirDNAzyme in vivo, we constructed CCl_4_‐induced fibrotic mice model and the treatment schedule was shown in **Figure** **6**A. After all treatments, the mice were executed, and then the serum and liver tissues were gathered to carry out series of experiments for further assessing the therapeutic effect. First, we determined the alanine transaminase (ALT) and aspartate transaminase (AST) activity in the serum (Figure 6B,C) to evaluate liver injury conditions. A vast increase of ALT and AST values after CCl_4_ induction was observed in comparison to healthy mice, indicating liver inflammation and damage. In contrast to other groups, P‐ZIF‐8‐cirDNAzyme treatment had distinctly decreased activity of ALT and AST, which illustrates that liver function was greatly improved. Inflammation is another critical factor governing hepatic stellate cells activation and fibrogenesis,^[^ ^26^ ^]^ and we then evaluated the effect of fibrosis therapy on inflammation. We tested proinflammatory cytokines in the serum that contribute to liver fibrogenesis. To our delight, the expressions of interleukin interleukin 1*β* (IL‐1*β)*, interleukin 6 (IL‐6), and transforming growth factor *β* (TGF‐*β)* were highly downregulated with P‐ZIF‐8‐cirDNAzyme treatment (Figure 6D–F). This indicated that P‐ZIF‐8‐cirDNAzyme could reduce the inflammatory response to a certain extent, which is beneficial to the remodeling of the collagen microenvironment and the recovery of liver function.

**Figure 7:**
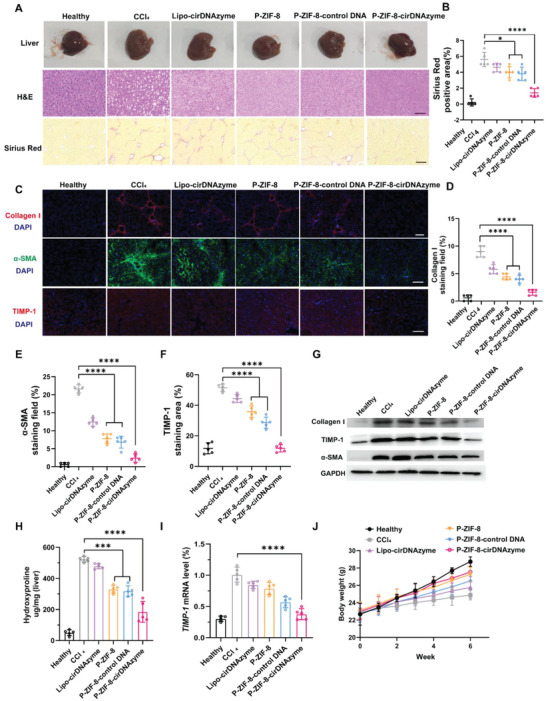
Histological analysis of livers of mice with CCl_4_‐induced liver fibrosis with different treatments. A) Representative images of liver photos, H&E (20×, Scale bar: 150 µm), and Sirius Red staining (10×, Scale bar: 300 µm) of liver tissue sections of mice with CCl_4_‐induced liver fibrosis with different treatments. B) Semiquantitative analysis of the area of Sirius Red staining sections. C) Immunofluorescence analysis of Collagen‐I, TIMP‐1, and *α*‐SMA protein of liver tissue sections of mice with CCl_4_‐induced liver fibrosis with different treatments (25×, Scale bar: 100 µm). D–F) Semiquantitative analysis of the (C). G) Western blot analysis of Collagen I and TIMP‐1 *α*‐SMA protein in the liver of CCl_4_‐induced fibrosis mice with various treatments. H) Hydroxyproline assay in livers of mice with CCl_4_‐induced liver fibrosis with various treatments (*n* = 5). I) RT‐qPCR assay for monitoring TIMP‐1 mRNA expression in livers of mice with CCl_4_‐induced liver fibrosis with various treatments (*n* = 5). J) The corresponding body weight changes of mice with CCl_4_‐induced liver fibrosis mice with various treatments (*n* = 5). All data are presented as the mean ± SD. The statistical significance was calculated via one‐way ANOVA with Tukey's post‐test (*P < 0.05, **P < 0.01, ***P < 0.001, and ****P < 0.0001; ns: no significance).

To further investigate the anti‐fibrotic activity in tissues, we performed histological analyses of liver tissue sections. The normal liver was smooth and reddish brown due to abundant blood, but the fibrotic liver swelled and was with muted color and rough surface (**Figure** **7**A). Next, liver micromorphology was examined with H&E staining and Sirius Red staining (Figure 7A). Livers from healthy mice showed a normal liver architecture, while livers from CCl_4_‐treated mice exhibited significant disruption of the liver architecture and large fibrous septa formation. In contrast, P‐ZIF‐8‐cirDNAzyme treated had lots of improvement than that of other groups. Similarly, P‐ZIF‐8‐cirDNAzyme treated has the least area of Sirius Red (Figure 7A,B), which indicated the least collagen. To corroborate the results, the immunofluorescence analyses were performed. We imaged *α*‐SMA, collagen I, and TIMP‐1 with fluorescent antibodies, respectively (Figure 7C). As expected, the P‐ZIF‐8‐cirDNAzyme group showed the lowest level of fibrotic protein expression (Figure 7D–F). Besides, analyses of protein expressions by Western blot obtained consistent results as well (Figure 7G). The level of collagen in P‐ZIF‐8‐cirDNAzyme was reduced by 73.12% which revealed that the collagen microenvironment was remodeled and meanwhile the *α*‐SMA and TIMP‐1 protein were also downregulated proving the reversal of liver fibrosis.

### 2.7 Anti‐Fibrotic Mechanism of P‐ZIF‐8‐cirDNAzyme In Vivo

To further explore the anti‐fibrosis mechanism, livers were ground and some biological indices were measured. First, hydroxyproline is a major component of collagen and comprises about 13.5% of the total amino acids in collagen.^[^ ^27^ ^]^ As previously explored in vitro, Zn (II) interference could inhibit the activity of P4H, thereby preventing the hydroxylation of collagen proline residues. Therefore, we tested the level of hydroxyproline in the liver to determine whether Zn (II) interference is effective in vivo (Figure 7H). Compared with CCl_4_ treated, Zn (II) contained nanoparticles (P‐ZIF‐8, P‐ZIF‐8‐control DNA, and P‐ZIF‐8‐cirDNAzyme) treatment greatly decreased the level of hydroxyproline, indicating that Zn (II) interference indeed could inhibit the hydroxylation of collagen proline residues in vivo. Notably, P‐ZIF‐8‐cirDNAzyme treated had the lowest level of hydroxyproline, which was highly possibly attributed to cirDNAzyme's function of degradation of existing collagen. Accordingly, ICP‐MS also proved the increase of Zn (II) content within aHSC after P‐ZIF‐8‐cirDNAzyme treatment (Figure S15, Supporting Information). Subsequently, RT‐qPCR analyzed that the TIMP‐1 mRNA was decreased to ≈38.3% in the P‐ZIF‐8‐cirDNAzyme group (Figure 7I), which proved cirDNAzyme's excellent function of gene silence in vivo. Surprisingly, the Zn (II) interference groups also showed the decreased TIMP‐1 mRNA, a possible explanation for this might be that the Zn (II) interference might cause complex changes in the intracellular signaling pathways, alleviating the pro‐fibrotic level, and then reduced the level of TIMP‐1 mRNA. This also indicated the mutual promotion between Zn (II) interference and TIMP‐1 gene silencing for anti‐liver fibrosis. During the treatment period, we also recorded the psychiatric condition and the body weights of mice in each group. The mice in the P‐ZIF‐8‐cirDNAzyme group showed the best living states and weight gain compared with other groups (Figure 7J). The above results suggested the outstanding therapeutic effect of P‐ZIF‐8‐cirDNAzyme on liver fibrosis.

## 3 Conclusion

In conclusion, we developed a biomimetic nano‐regulator (P‐ZIF‐8‐cirDNAzyme) for precisely and efficiently regulating aHSC to remodel collagen microenvironment. We demonstrated that the nanoparticles with platelet membrane coating could escape the clearance of the mononuclear scavenger system and move toward the inflammatory fibrotic liver, significantly enhancing the accumulation of the nanoparticles in the liver. Furthermore, the platelet membrane surface protein CD62p can specifically bind to the CD44 receptor overexpressed on aHSC. This strategy indicated platelet membrane may act as an attractive material for precisely targeting aHSC. The accumulation of Zinc ions in aHSC significantly inhibited collagen synthesis via ion interference. Meanwhile, the circular DNAzyme exhibited high stability and downregulated TIMP‐1 mRNA expression in aHSC, greatly promoting the degradation of collagen. Consequently, P‐ZIF‐8‐cirDNAzyme recovered the balance between collagen deposition and degradation, and restored liver function nearly to normal. Besides, it showed a broad influence on multiple fibrosis–related signaling pathways (Wnt, TGF‐*β*, and PI3K‐AKT). Overall, this work represents a fascinating therapeutic platform enabling ion interference and gene silencing, collectively acting in aHSC for the reversal of liver fibrosis, which provides a new idea and paradigm for delaying the progression of liver disease.

## 4 Experimental Section

### Materials

Zinc nitrate hexahydrate (Zn(NO_3_)_2_·6H_2_O), 2,4‐dimethylimidazole 2 and enzyme collagenase were purchased from Sigma‐Aldrich (St. Louis, MO, USA). T4 DNA ligase and DNase I (Catalog no. E127‐01A) were purchased from Novoprotein Scientific (China). Zinquin AM (Catalog no. HY‐D1441) was purchased from MedChemExpress (MCE) company. TPEN (Catalog no. N159625) was purchased from Aladdin. P4H activity assay kit (Catalog no. QYK‐17562) was purchased from Qiyuan Biotechnology (Shanghai, China). Lyso‐Tracker Red and Hoechst 33 342 (100 ×) were purchased from Yeasen Biotechnology (Shanghai) Co., Ltd. Bicinchoninic acid protein assay kit and D‐PBS (Catalog no. D1040) were purchased from Beijing Solarbio Science and Technology Co., Ltd. (Beijing, China). Anti‐CD41 (Catalog no. 24552‐1AP), anti‐CD47 (Catalog no. 66304‐1‐AP), anti‐CD62p (Catalog no. 60322‐1‐Ig), anti‐GAPDH antibody (Catalog no. 600041‐Ig), and goat anti‐rabbit IgG secondary (Catalog no. 60004–1‐Ig) were purchased from Proteintech (China). Anti‐TIMP‐1 antibody (Catalog no. bsm‐10895 M) was purchased from Beijing Biosscn Biotechnology Co., Ltd. Anti‐Collagen I monoclonal antibody (Catalog no. ab270993) and anti‐*α*‐SMA monoclonal antibody (Catalog no. ab124964) were purchased from Abcam (Cambridge, UK). Goat anti‐mouse IgG secondary antibody conjugated with Cy3 (Catalog no. SA00009‐1) were purchased from Proteintech (China). DMEM/F12, HEPES, AML‐12 cell specific culture‐medium (Catalog no. CM‐0602) and RPMI‐1640 were purchased from Procell Life Science and Technology Co., Ltd. Cell Counting Kit‐8 (CCK‐8, Catalog no. K1018) was purchased from Beyotime Biotech. Inc. (Shanghai, China). Lipofectamine 2000 Transfection Reagent (Catalog no. 11 668 019) was purchased from Thermo Fisher. Thrombin (Catalog no. JL45766), ADP (Catalog no. JL20324), and TXB2 (Catalog no. JL11127) ELISA kits were purchased from Shanghai Jianglai industrial Limited By Share Ltd. Cy5‐DNAzyme and DNA substrate were purchased from Tsingke Biotech Co., Ltd. GelRed Nucleic acid dye (Catalog no. TSJ003‐10) was purchased from Tsingke Biotech Co., Ltd. DEPC water was purchased from Sangon Biotech, Shang hai, China. TRIzon Reagent (Catalog no. CW0580S), 6× DNA Loading Buffer, and 1 kb DNA Marker were purchased from JiangSu CoWin Biotech (CWBIO). HiScript II 1st Strand cDNA Synthesis Kit (Catalog no. R212‐01) was purchased from Nanjing Vazyme Biotech Co., Ltd. qPCR assay kit (Catalog no. AQ601‐01) was purchased from TransGen Biotech in Beijing. Hydroxyproline assay kit and ELISA kit (IL‐6, IL‐1*β*, TGF‐*β*, and TNF‐*α*) were purchased from Shanghai Enzyme‐linked Biotechnology.

### Instruments

TEM images were obtained by a TEM (JEM 1200EX, JEOL, Japan). XRD patterns were gained from X‐ray power diffractometer (D8 Advance, Bruker, Germany) in the range of 5–50° (2*θ*). Size distribution and zeta potential were measured by a Zeta sizer (Nano ZS‐90, Malvern, UK). The UV–vis spectrum was obtained using a UV–vis spectrometer (UV2700, Shimadzu, Japan). TGA profiles were acquired using a gravimetric analyzer (Q500, TA, USA). The cell viability was measured using a microplate reader (Synergy H1, BioTek, USA). The fluorescence images were obtained from a laser scanning confocal microscopy (TCS SP8, Leica, Germany). ICP‐MS results were acquired from an inductively coupled plasma mass spectrometer (7500ce, Agilent, USA). In vivo biodistribution was detected using a small animal imaging system (Xtreme, Bruker, Germany). Blood biochemistry analysis was conducted by an Auto biochemical analyzer (Chemray 240, Rayto, China). Blood routine was examined by Auto Hematology Analyzer (BC‐2800vet, Mindray, China).

### Derivation of Platelet Membrane Vesicles

To obtain platelet membrane vesicles, the fresh mice derived platelets collected into heparin sodium‐treated blood collection tubes were isolated via an abdominal aortic method. To separate white blood cells and red blood cells, the collected fresh blood samples were centrifuged twice at 100 g for 20 min at 25 °C. To avoid platelet activation, the purified platelet‐rich plasma was diluted with PBS buffer containing 1 mm PMSF and 2 µm prostaglandin E1. Subsequently, solutions were then centrifugated at 800 g for 20 min at room temperature for pelleting platelets, after which the obtained precipitate was resuspended in PBS with 1 mm EDTA and PMSF. 1.5 mL PBS almost containing platelets were resuspended in 1.5 mL PBS for platelet membrane extraction. A repeated freeze‐thaw process was conducted for platelet membrane derivation. First, platelet suspensions were frozen at −80 °C, thawed at room temperature, and centrifugated at 4000 g for 3 min. The obtained sediment was washed with PBS containing 1 mm PMSF, then sonicated for 5 min by sonicator at 42 kHz, 100 W. Morphological examination by TEM was used for verifying the presence of platelet membrane vesicles. The concentration of extracted platelet was measured using Nanoparticle Tracking Analysis.

### Synthesis of cirDNAzyme

Well‐designed DNAzyme and DNAzyme′ undergone annealing reaction in T4 buffer and were incubated with T4 DNA ligase at room temperature for 12 h to acquire the TIMP‐1 cirDNAzyme. Finally, it was successfully prepared by PAGE analysis. DNAzyme or T4 DNA ligase untreated sequences of hybridization were degraded under Exo‐I, while cirDNAzyme had stability of anti‐Exo‐I. The design referred to the previous work.

### Synthesis of ZIF‐8, ZIF‐8‐cirDNAzyme, and P‐ZIF‐8‐cirDNAzyme Nanoparticles

0.5 mL Zn (NO_3_)_2_·6H_2_O (30 mg mL^−1^) and 40 µL of cirDNAzyme (100 µm) were dissolved in deionized water (0.5 mL). 1 mL of 2‐methylimidazole (33 mg mL^−1^) was dissolved in methanol. Under stirring, the aqueous zinc nitrate and cirDNAzyme were added into the solution of 2‐methylimidazole and were incubated for 5 min. The product was collected by centrifugation and washed three times with a mixture of H_2_O and ethanol. ZIF‐8‐cirDNAzyme nanoparticles were obtained by vacuum drying. ZIF‐8 nanoparticles were synthesized by the similar method. P‐ZIF‐8‐cirDNAzyme was prepared by adding platelet membrane vesicles aqueous solution to the above products and the mixture was sonicated for 10 min. The obtained P‐ZIF‐8‐cirDNAzyme nanoparticles were dried in a vacuum.

### In Vitro Zn (II) Release from P‐ZIF‐8‐cirDNAzyme

P‐ZIF‐8‐cirDNAzyme nanoparticle (100 µg mL^−1^) was sufficiently dissolved into different pH (7.4, 6.8, and 5.0) for different time points, respectively. Subsequently, the mixture was centrifuged at 12 000 rpm for 20 min, the supernatant was collected for detecting Zn (II) concentrations by ICP‐MS. Meanwhile, sediment was utilized to examine the change of UV absorbance from 200 to 400 nm by a UV–vis spectrometer (UV2700, Shimadzu, Japan).

### In Vitro cirDNAzyme Release from P‐ZIF‐8‐cirDNAzyme

P‐ZIF‐8‐cirDNAzymes (cirDNAzyme was labeled with Cy5) were incubated in pH 7.4, 6.8, and 5.5 at 37 °C, 150 rpm, and lucifuge environment for different time points. Subsequently, the mixture was centrifuged at 12 000 rpm for 20 min, the supernatant was collected and fluorescence intensity was measured by microplate reader (Ex: 638, Em:658). The DNA concentration was calculated by fluorescence intensity according to a standard curve of free Cy5‐cirDNAzyme.

### Cleavage Ability of cirDNAzyme and P‐ZIF‐8‐cirDNAzyme In Vitro

The experiment of cirDNAzyme cleavage in vitro was carried out: cirDNAzyme (500 nm) was incubated with an equal amount of TIMP‐1 substrate (500 nm) (Table S2, Supporting Information) at room temperature for 2 h in tris‐HCl buffer (pH = 7.4) containing different concentrations of Zn (II) (50, 100, 200, and 500 µm). After incubation, the cleavage efficiency was verified by polyacrylamide gel electrophoresis. P‐ZIF‐8‐cirDNAzyme (100 µg mL^−1^) and TIMP‐1 substrate (500 nm) were incubated at different pH (7.4, 6.8, and 5.0) for 4 h. The cleavage efficiency was verified by polyacrylamide gel electrophoresis.

### Stability of cirDNAzyme and P‐ZIF‐8‐cirDNAzyme In Vitro

The experiment of cirDNAzyme stability in vitro was carried out: DNAzyme, DNAzyme′, and cirDNAzyme (with and without T4 DNA ligase) were incubated with Exo‐I at 37 °C for 2 h in PBS buffer and then the stability was analyzed by agarose gel electrophoresis. The experiment of P‐ZIF‐8‐cirDNAzyme stability in vitro was carried out: DNAzyme and P‐ZIF‐8‐cirDNAzyme with equivalent amount of DNAzyme were incubated with DNase I at 37 °C for 0, 10, 30, and 60 min, and then the stability was analyzed by PAGE.

### Molecular Simulations Characterize the Binding Mode of P4H and Substrate Peptide in Fe^2+^ and Zn (II) System

The P4H protein used in this study was encoded by mouse prolyl 4 hydroxylase subunit alpha‐1 gene (UniProt number Q60715). The 3D structure of P4H protein was downloaded from Alpha Fold 2 database under the number AF‐Q60715‐F1. Due to the high homology of the P4H protein, the crystal structure of the viral collagen prolyl hydroxylase (PDB number 5C5T) was used as the template to construct the cofactor metal iron ion (Fe^2+^), 2‐oxoglutrate (2‐OG), and its catalytic substrate peptide complex system 1. To investigate the effect of Zn (II) on the catalytic activity of P4H, system 2 was constructed in this study, and the difference from the initial results of the system is that Fe^2+^ was replaced by Zn (II), and all structures were energy minimized under the Amber 99SB force field. The molecular simulation experiment was commissioned by Phad Calculation (Chengdu, China.)

### MM/GBSA Analysis of Δ*G* of P4H and Substrate Peptide in Fe^2+^ and Zn (II) System

To investigate the effect of Zn (II) on the ability of P4H to catalyze prolyl‐containing peptides, the binding capacity of P4H to the catalytic substrate peptides in the presence of cofactors Fe^2+^ and Zn (II) was calculated. The binding free energy Δ*G*_bind_ of protein P4H and polypeptide substrate was calculated using the MM/GBSA. The specific method is described as follows. The binding energy was calculated according to the following formula using the program gmx‐MMPBSA according to the kinetic simulation trajectory file

(1) $$\Delta G_{\mathrm{bind}}=\Delta H-T\Delta S\approx \Delta G_{\mathrm{solv}}+\Delta G_{\mathrm{GAS}}-T\Delta S$$

(2) $$\Delta G_{\mathrm{GAS}}=\Delta E_{\mathrm{int}}+\Delta E_{\mathrm{vdw}}+\Delta E_{\mathrm{ele}}$$

(3) $$\Delta G_{\mathrm{solv}}=\Delta E_{\mathrm{SURF}}+\Delta E_{\mathrm{GB}}$$

Δ*G*_GAS_ is the kinetic energy difference between receptor and ligand before and after binding in vacuum, which is further subdivided into *E*_int_, *E*_vdw_, and *E*_ele_, where *E*_int_ represents the energy change of bond, bond angle, and dihedral angle, *E*_vdw_ is the van der Waals energy change before and after binding, and Δ*E*_ele_ is the electrostatic interaction change. Δ*G*_solv_ is a solvent effect term, which is divided into polar term Δ*E*_GB_ and non‐polar term Δ*E*_SURF_. The calculation of Δ*E*_GB_ was relatively complex and time‐consuming, so APBS program was used to calculate this part. ∆*E*_SURF_ was calculated by solution accessible surface area, where *γ* = 0.0227 kJ (mol nm^2^)^−1^, *β* = 3.85 kJ mol^−1^. *T*Δ*S* is the entropy change, which was the most time‐consuming and difficult to calculate. At present, the calculation accuracy of this term is low, and this energy had little influence on the contribution of binding free energy. Therefore, this term was regarded as 0 when calculating the binding free energy.

### Cell Lines and Cell Culture

HSCT6 cells were purchased from the Kunming Cell Bank of Chinese Academy of Sciences (Yunnan, Kunming, China). Raw 264.7 cells were purchased from Cell Bank of Chinese Academy of Science (Shanghai, China). HSCT6 cells were maintained DMEM/F‐12 supplemented with 10% FBS (Gibco). Raw 264.7 cells were maintained in DMEM (high glucose medium) with 15% FBS (Biological Industries). All cells were cultured at 37 °C in a humidified incubator containing 5% CO_2_ and all experiments were conducted on cells in the exponential growth phase.

### Cellular Uptake Assay

HSCT6, Raw 264.7, and AML‐12 cells were seeded at 35 mm confocal dish at 1 × 10^5^ per dish and cultured for 12 h. After that, cells were washed with PBS for three times and incubated with Cy5‐cirDNAzyme, ZIF‐8‐Cy5‐cirDNAzyme, and P‐ZIF‐8‐Cy5‐cirDNAzyme (10 µg mL^−1^) for 4 h. For anti‐CD62p/anti‐CD44 antibody block group, first anti‐CD62p antibody was incubated with P‐ZIF‐8‐cirDNAzyme or anti‐CD44 antibody was incubated with HSCT6 cells for 2 h. Then the pre‐treated P‐ZIF‐8‐cirDNAzymes were incubated with HSCT6 cells for 4 h. After incubation, cells were washed with PBS three times and stained with 1 mL Hoechst 33342 for 10 min. After staining, images were acquired using a Leica laser scanning confocal microscope (TCS SP8, Leica, Germany). Last, the cells were collected by trypsin digestion. The fluorescence intensity of collected cells was detected by a flow cytometer (BD Accuri®C6, American).

### Lysosome Escape Assay

Cells were seeded at a 35 mm confocal dish at 1 × 10^5^ per dish and cultured for 12 h. After that, cells were washed with PBS for three times and incubated with P‐ZIF‐8‐Cy5‐cirDNAzyme (10 µg mL^−1^) for 0.5, 1, 2, and 4 h. After incubation, cells were washed with PBS for three times and incubated with Lyso‐Tracker Red (75 nm) for 15 min. Then cells were washed with PBS for three times and stained with 1 mL Hoechst 33342 for 10 min. After staining, images were acquired and the person correlation coefficients were analyzed by a Leica laser scanning confocal microscope (TCS SP8, Leica, Germany).

### Intracellular Free Zn (II) Detection

To dectect the intracellular free Zn (II) accumulation by P‐ZIF‐8‐cirDNAzyme (10 µg mL^−1^), cells were seeded at 35 mm confocal dish at 1 × 10^5^ per dish and cultured for 12 h. After that, cells were washed with PBS for three times and incubated with P‐ZIF‐8‐cirDNAzyme (10 µg mL^−1^) for 4 h. For TPEN group, HSCT6 cells were first incubated with 10 µg mL^−1^ P‐ZIF‐8‐cirDNAzyme for 4 h and then treated with 1 µm TPEN for 0.5 h. After incubation, cells were washed with PBS for three times. Subsequently, the Zinquin (50 µm, 30 min) were incubated with cells for indicating intracellular free Zn (II) accumulation. Last, a Leica laser scanning confocal microscope was utilized for characterizing fluorescence intensity.

### Quantitative Analysis of Intracellular Free Zn (II) Levels

Cells were seeded at a 35 mm confocal dish at 1 × 10^5^ per dish and cultured for 12 h. After that, cells were washed with PBS for three times and incubated with P‐ZIF‐8‐cirDNAzyme (10 µg mL^−1^) for 4 h. After incubation, cells were washed with PBS for three times and collected by trypsin digestion. The collected cells were resolved by nitric acid and hydrogen peroxide (30%, 3 mL). The mixture was added with deionized water to 10 mL. The final solutions were used to measure the concentration of Zn (II) by ICP‐MS.

### TIMP‐1 Measurement via RT‐qPCR

Cells were seeded at 6‐well plates at 1 × 10^5^ per well and cultured for 12 h, and then were incubated with 10 µg mL^−1^ of different nanoparticles for 24 h. After incubation, precipitate of cells was completely collected by trypsin digestion and centrifugation (1000 rpm, 5 min). Then mRNA was isolated with TRIzol Reagent kit. 2 µg of RNA sample was utilized to obtain cDNA. 2 µL of cDNA sample and specific primers (Table S3, Supporting Information) were used to amplify TIMP‐1 cDNA according to the manufacturer's parameters (qPCR Quantitation Kit) on a real‐time PCR machine.

### CD44, TIMP‐1, *α*‐SMA, and Collagen I Measurement via Western Blot

Cells were seeded at 6‐well plates at 1 × 10^5^ per well and cultured for 12 h, and then were incubated with 10 µg mL^−1^ of different nanoparticles for 24 h. After incubation, precipitate of cells was completely collected by trypsin digestion and centrifugation (1000 rpm, 5 min) and washed twice with PBS. Then RIPA and PMSF were used to Crack cells. The protein content was measured by Bicinchoninic Acid Protein quantification kit. Western blot analysis was conducted using standard method and the GAPDH was used as internal references.

### Collagen I Measurement via Immunofluorescence

Cells were seeded at 6‐well plates at 1 × 10^5^ per well and cultured for 12 h, and then were incubated with 10 µg mL^−1^ of different nanoparticles for 24 h. After incubation, cells were fixed with 4% paraformaldehyde for 15 min at room temperature. Then after permeabilized and closured, they were incubated with collagen antibody at 4 °C overnight. Afterward, they were stained with fluorescent secondary antibody. After staining, images were acquired using a fluorescence microscope.

### Cytotoxicity Assay

The in vitro cytotoxicity assay of P‐ZIF‐8‐cirDNAzyme was conducted with CCK8 assay following the literature procedure. HSCT6 cells were seeded in 96‐well plates at 1 × 10^4^ per well and cultured in 100 µL fresh complete medium for 12 h. Another complete medium containing P‐ZIF‐8‐cirDNAzyme (0,2, 4, 6, 8, and 10 µg mL^−1^) were added, respectively. After incubating for 24 h, 10 µL CCK8 was added into the medium and kept for another 1 h. The optical density (OD) was measured at 450 nm by a microplate reader (Synergy H1, American). Cell viability of different nanoparticles was calculated by the following formula: cell viability (%) = (OD_sample_ − OD_blank_/OD_control_ − OD_blank_) × 100%.

### RNA‐Seq for Transcriptome Analysis

HSCT6 cells were seeded at 6‐well plates at 1 × 10^5^ per well and cultured for 12 h, and then were incubated with 10 µg mL^−1^ P‐ZIF‐8‐cirDNAzyme for 24 h. Untreated HSCT6 cells were used as control group. Each group had three parallel replicates. After P‐ZIF‐8‐cirDNAzyme incubation, cells were washed, digested, and centrifugally collected for transcriptome analysis. RNA was extracted with Trizol Reagent kit. RNA sequencing and bioinformatic data collection were performed by Majorbio (Shanghai, China).

### Animal Experiments

C57BL/6J mice (20 ± 2 g, male) were purchased from Hunan SJA Laboratory Animal Company, and fed at the condition of 25 ± 2 °C and 55% humidity with 12 h light/dark cycle. The license number is SCXK (xiang) 2019‐0004. All the animal experiments were performed in accordance with the guidelines of the Regional Ethics Committee for Animal Experiments and the Care Regulations approved by the Institutional Animal Care and Use Committee of Zhengzhou University. The animal laboratory's accreditation number is 110 322 211 102 955 054. Mice were first randomly divided into groups containing at least five mice per group. Then, CCl_4_, diluted in mineral oil (1:3), was given by intraperitoneal (i.p.) injection two times a week for 6 weeks (4 µLg^−1^ body weight); mice in the nonfibrotic control group received oil alone.

### Serum Index Assay

The CCl_4_ induced liver fibrosis mice were divided into five groups. The different formulations (P‐ZIF‐8‐cirDNAzyme 10 µg mL^−1^) were intravenously injected once a day for 3 days. The blood of mice was collected after injection 24 h. The samples were utilized to examine those parameters of blood biochemistry and blood routine by Auto biochemical analyzer (Chemray 240, Rayto, Shenzhen) and Auto Hematology Analyzer (BC‐2800vet, Mindray, China), respectively.

### Isolation of Activated Hepatic Stellate Cells

Activated hepatic stellate cells were obtained by in situ liver perfusion and density gradient centrifugation. The specific operation is as follows. After the limbs were fixed, 75% alcohol was sprayed to disinfect the chest and abdomen. The abdomen was cut through a wedge to fully expose the subhepatic inferior vena cava and portal vein. The suprahepatic inferior vena cava was clamped, the portal vein was punctured with a 24 G vein trocar, and the fixed cannula was ligated. Hank's solution without calcium and magnesium ions was infused, and the infrahepatic inferior vena cava was cut as the outflow tract. The hearts were punctured and the mice were sacrificed, then 1 mL of collagenase was injected into a 24 G venous cannula. The portal ligament and falciform ligament were cut, and the liver was removed intact and placed in Hank's solution without calcium and magnesium ions at 4 °C. The liver was ground into small pieces (diameter < 1 mm), then 9 mL collagenase was added and poured into a small beaker and sealed. The digestion was carried out in a water bath at 37 °C for 30 min with shaking. Then RPMI‐1640 medium containing serum at 4 °C was added and diluted for 2 min with stirring. The 100 µm nylon strainer was used to filter out undigested tissue. The cells were washed three times with Hank's solution without calcium and magnesium ions. The first and third times were centrifuged at 1700 r min^−1^ at 4 °C for 6 min, and the supernatant was discarded. After a second centrifugation at 400 r min^−1^ for 5 min at 4 °C, the supernatant was aspirated. The precipitate was resuspended with 1 mL Percoll and then added to the top layer of 8 mL 60% Percoll gradient centrifugation solution for centrifugation at 4 °C and 9600 r min^−1^ for 21 min. After centrifugation, the top layer of the liquid was the yellow‐white hepatocyte debris layer, and the lower layer of white material was the aHSC layer. This layer of material was carefully sucked through a straw, resuspended to 50 mL, and washed twice with RPMI‐1640 supplemented with antibiotics at 4 °C, 1700 r min^−1^, centrifuged for 6 min, and the precipitate was the aHSC.

### In Vivo Biodistribution

For in vivo imaging studies, free Cy5‐cirDNAzyme, ZIF‐8‐Cy5‐cirDNAzyme, or P‐ZIF‐8‐Cy5‐cirDNAzyme (1 mg kg^−1^ body weight) were injected into fibrotic mice at different times via the tail vein. Mice were anesthetized and their hairs were removed through the shaving device. The mice were imaged 4, 8, 12, 24, and 48 h later using an in vivo imaging system (FX‐Pro; Bruker). Corresponding ex vivo imaging assays were also performed using excised organs.

### Colocalization of cirDNAzyme and Activated HSCs in Fibrotic Liver

For localization studies, ZIF‐8‐Cy5‐cirDNAzyme and P‐ZIF‐8‐Cy5‐cirDNAzyme (1 mg kg^−1^) were injected into fibrotic mice via the tail vein. Twenty‐four hours after the injection, mice were sacrificed and liver tissue was harvested and frozen‐sectioned. Sections were then stained with a rabbit anti‐mouse *α*‐SMA antibody (Abcam, UK) at 4 °C overnight to label HSCs. Sections were next incubated with DyLight 488‐labeled goat antirabbit secondary antibody 37 °C for 45 min, followed by staining of nuclei with 4,6‐diamidino‐2‐phenylindole (DAPI; Sigma‐Aldrich). Finally, frozen sections were imaged with a MIDI Slide scanner (3D HISTECH, Hungary).

### In Vivo Anti‐Fibrosis Efficiency

Analysis of serum enzymes were as follows: Serum levels of the liver injury markers, ALT and AST in various animal groups were examined by Auto biochemical analyzer (Chemray 240, Rayto, Shenzhen). Histological analysis was as follows: Paraffin‐embedded liver tissues were sectioned at 5 µm, then deparaffinized and hydrated. Thereafter, sections were stained with a H&E staining Kit (Nanjing, China) or with 0.1% w/v Sirius Red (Direct Red 80; Sigma‐Aldrich) in a saturated aqueous solution of picric acid for 1 h. Slides were then rinsed twice in 0.01 n HCl for 15 min each to remove unbound dye. After dehydration, slides were mounted and photographed at 20× and 40× magnifications.

### Statistical Analysis

All studies were evaluated in at least three independent experiments for each condition to ensure reproducibility. Data were expressed as mean ± SD. Significant differences between different groups were determined using two‐tailed unpaired Student's *t*‐tests for two‐group comparisons and one‐way analysis of variance (ANOVA) with post hoc Tukey's test for multiple‐group comparisons (*P < 0.05, **P < 0.01, ***P < 0.001 and ****P < 0.0001; ns: no significance). Statistical analyses were performed using IBM SPSS Statistics 26 software.

## Conflict of Interest

The authors declare no conflict of interest.

## Supporting information

Supporting Information

## Data Availability

The data that support the findings of this study are available in the supplementary material of this article.
