# Supplementary material for: Remodeling Collagen Microenvironment in Liver Using a Biomimetic Nano‐Regulator for Reversal of Liver Fibrosis
Source: Adv Sci (Weinh). 2023 Apr 23;10(18):2300127. doi: 10.1002/advs.202300127 (PMC10288244; doi:10.1002/advs.202300127)
Supplement: Supplementary file 1 — Supporting Information [file ADVS-10-2300127-s001.pdf]

Supporting Information

Remodeling Collagen Microenvironment in Liver Using a Biomimetic Nano-Regulator for Reversal of Liver Fibrosis

Yan Liang,[#] *Jinjin Wang,*[#] *Chenlu Xu, Wenshuai Han, Sixuan Wu, Yonghua Wu, Jingge Zhang, Junjie Liu, Zhenzhong Zhang,* Jinjin Shi,* Kaixiang Zhang**

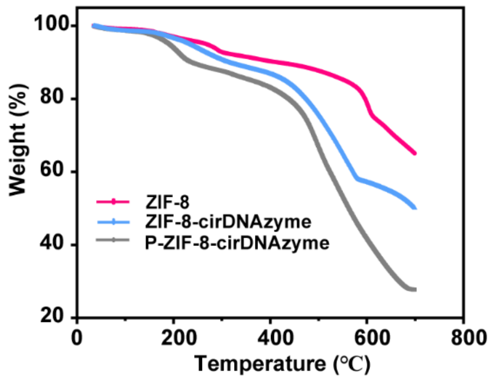

Figure S1. TGA analysis of ZIF-8, ZIF-8-cirDNAzyme and P-ZIF-8-cirDNAzyme. The thermal gravimetric analysis (TGA) was carried out on ZIF-8, ZIF-8-cirDNAzyme and P-ZIF-8-cirDNAzyme. The deeper drop of weight in P-ZIF-8-cirDNAzyme revealed the encapsulation of cirDNAzyme and the modification with the platelet membrane.

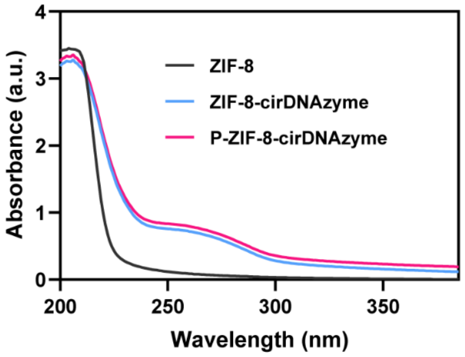

Figure S2. UV-vis spectrums analysis of the solutions containing ZIF-8, ZIF-8-cirDNAzyme, and P-ZIF-8-cirDNAzyme. UV-vis absorbance spectra of ZIF-8-cirDNAzyme and P-ZIF-8-cirDNAzyme revealed a characteristic absorption of cirDNAzyme at 260 nm, which was not observed in pure ZIF-8, indicating that cirDNAzymes were successfully encapsulated in ZIF-8-cirDNAzyme and P-ZIF-8-cirDNAzyme.

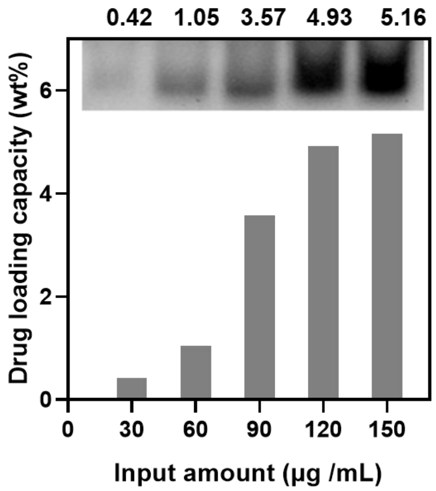

Figure S3. Analysis of loading efficiency under different input amounts of cirDNAzyme by agarose gel electrophoresis. With the increasing input amount of cirDNAzyme, the higher loading capacity of cirDNAzyme into P-ZIF-8-cirDNAzyme was obtained as the agarose gel electrophoresis showed.

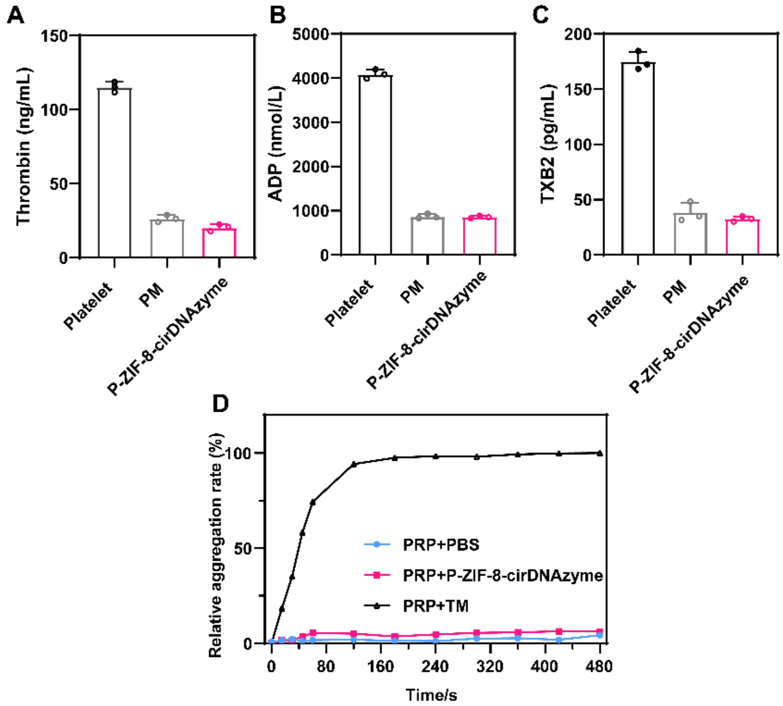

Figure S4. Platelet-activating contents analysis. The content of thrombin A), adenosine diphosphate (ADP) B), and thromboxane B2 (TXB2) C) in platelets. Platelet membrane (PM) and P-ZIF-8-cirDNAzyme were quantified by the corresponding ELISA detection kit (n=3). D) Platelet aggregation analysis by incubating EDTA-stabilized platelet-rich plasma (PRP) with PBS, P-ZIF-8-cirDNAzyme, or thrombin, respectively, followed by examining spectroscopic solution turbidity (n=3). The result verified the thrombin, ADP, and TXB2 were effectively removed from PM and P-ZIF-8-cirDNAzyme, thereby

preventing P-ZIF-8-cirDNAzyme from the thrombotic response, which provides a reassuring pill for safe application of P-ZIF-8-cirDNAzyme in vivo. Data are presented as mean ± SD (n=3).

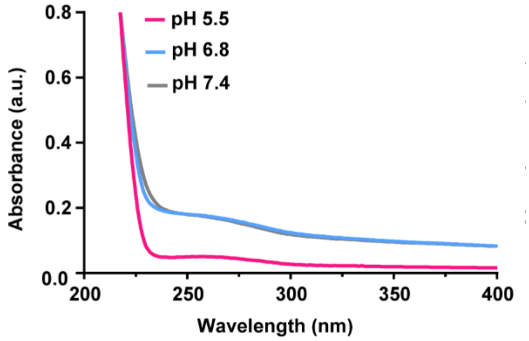

Figure S5. UV-vis absorbance spectra of P-ZIF-8-cirDNAzyme under different pH solution. UV-vis analysis was carried out for evaluating the pH-responsive ability of P-ZIF-8-cirDNAzyme. The decline of absorbance was observed from pH 7.4 to pH 5.5, demonstrating the ZIF-8-based skeleton collapse of P-ZIF-8-cirDNAzyme under acid conditions.

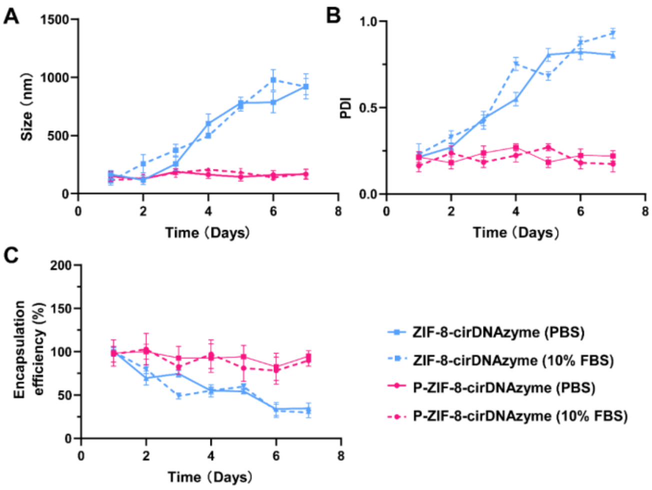

Figure S6. Stability of ZIF-8-cirDNAzyme and P-ZIF-8-cirDNAzyme in PBS and 10%FBS. Data are presented as mean ± SD (n=3). ZIF-8-cirDNAzyme was unstable both in PBS and 10% FBS. In contrast, P-ZIF-8-cirDNAzyme contains a stability size within seven days.

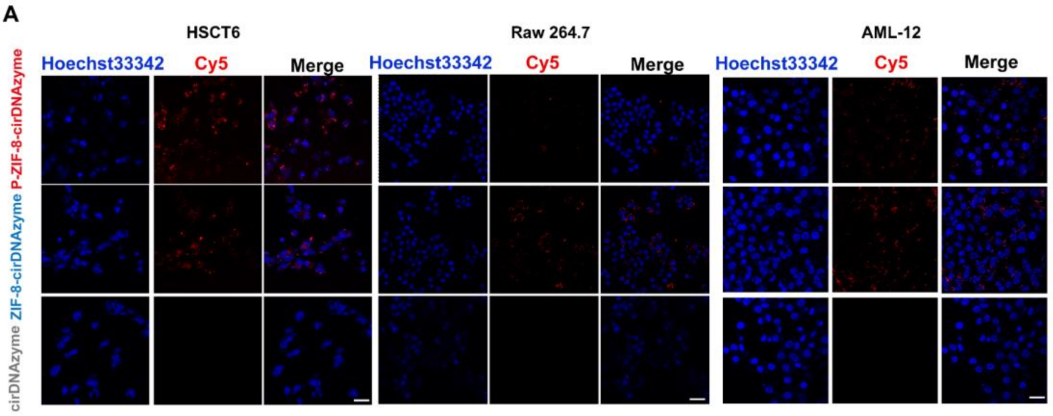

Figure S7. The different cell uptake of P-ZIF-8-cirDNAzyme, ZIF-8-cirDNAzyme and cirDNAzyme. CLSM images of HSCT6, Raw 264.7 and AML-12 cells treated with free cirDNAzyme, ZIF-8-cirDNAzyme and P-ZIF-8-cirDNAzyme, (Cy5: red fluorescence), Scale bar: 25 μm.

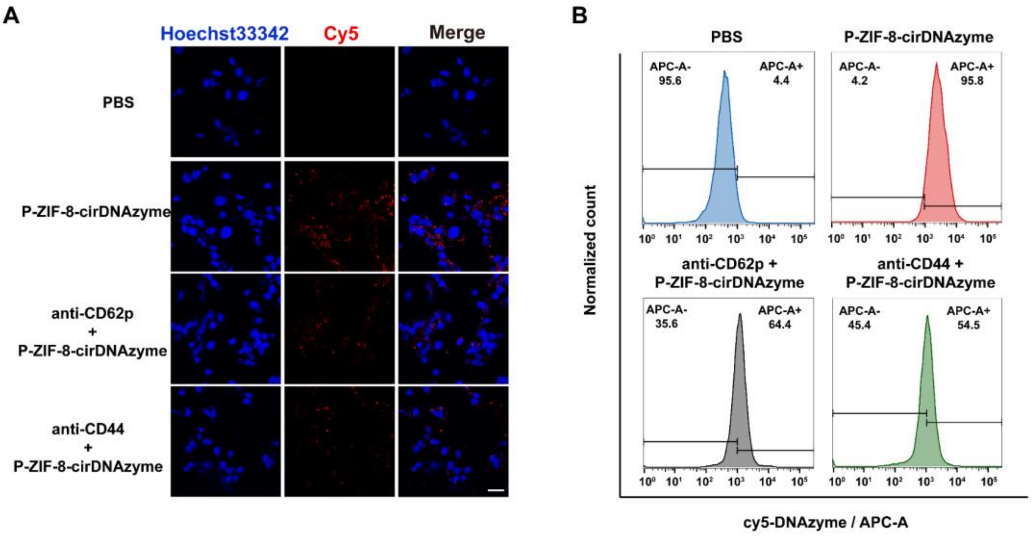

Figure S8. CLSM images A) and Flow cytometer analysis B) of HSCT6 cells internalization of P-ZIF-8-cirDNAzyme pretreated with antibody. Scale bar: 25 μm.

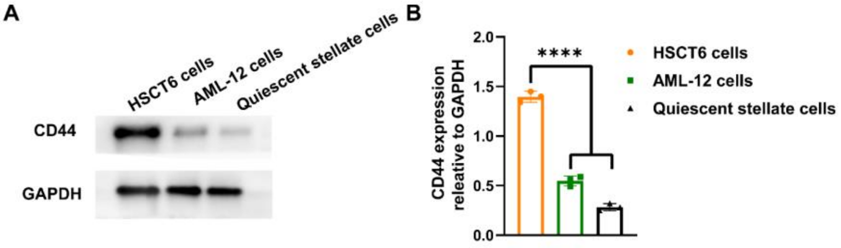

Figure S9. Western Blot assay A) and Semi-quantitative analysis B) of CD44 expression in HSCT6, AML-12 and Quiescent stellate cells. Data are presented as mean ± SD (n=3). The statistical significance was calculated via one-way ANOVA

with Tukey's post-test (*****P* < 0.0001).

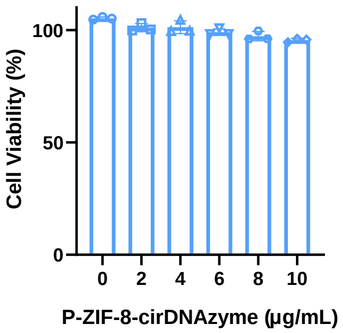

Figure S10. Cytotoxic assay of AML-12 cells subjected to different concentrations of P-ZIF-8-cirDNAzyme (n=3). The data are presented as the mean ± SD. There was no obvious toxic to hepatocytes (AML-12) at the preparation concentration of 0-10 μg/mL.

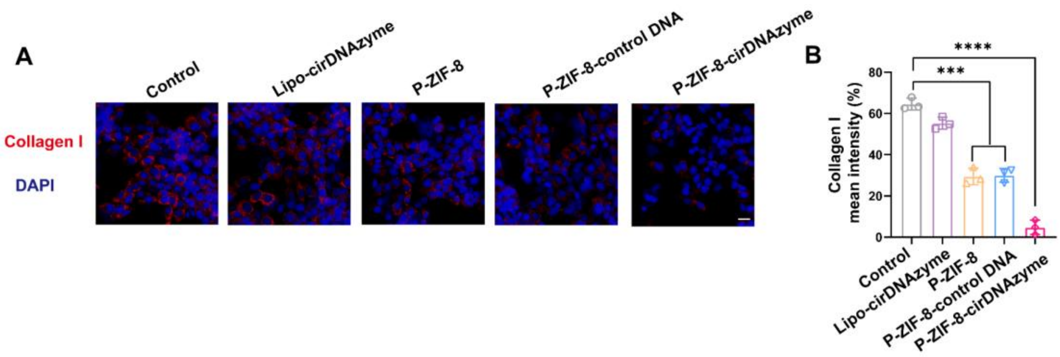

Figure S11. A) Immunofluorescence analysis of Collagen I protein of HSCT6 cells with different treatments, Scale bar: 40 μm. B) Semi-quantification of mean fluorescence intensity of A), n=3. The data are presented as the mean ± SD. The statistical significance was calculated via one-way ANOVA with Tukey's post-test (****P* < 0.001 and *****P* < 0.0001).

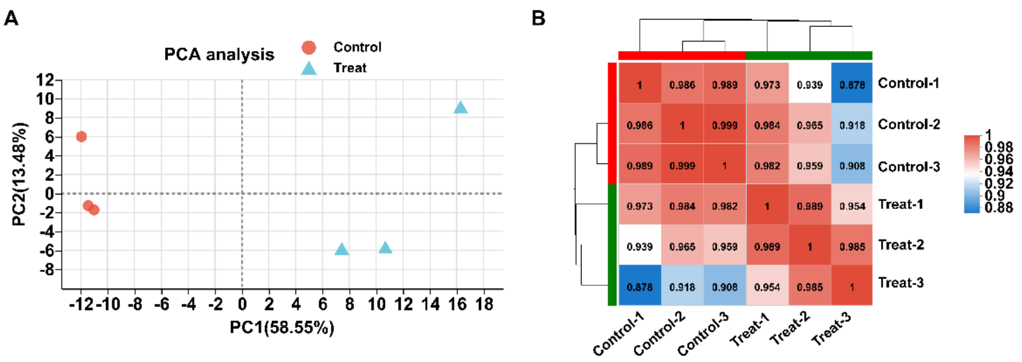

Figure S12. A) The diagram of principal component analysis (PCA). The abscissa is the first principal component and the ordinate is the second principal component. B) Heat map of inter-sample correlation. The horizontal and vertical coordinates in the figure are the square of the correlation coefficients of each sample.

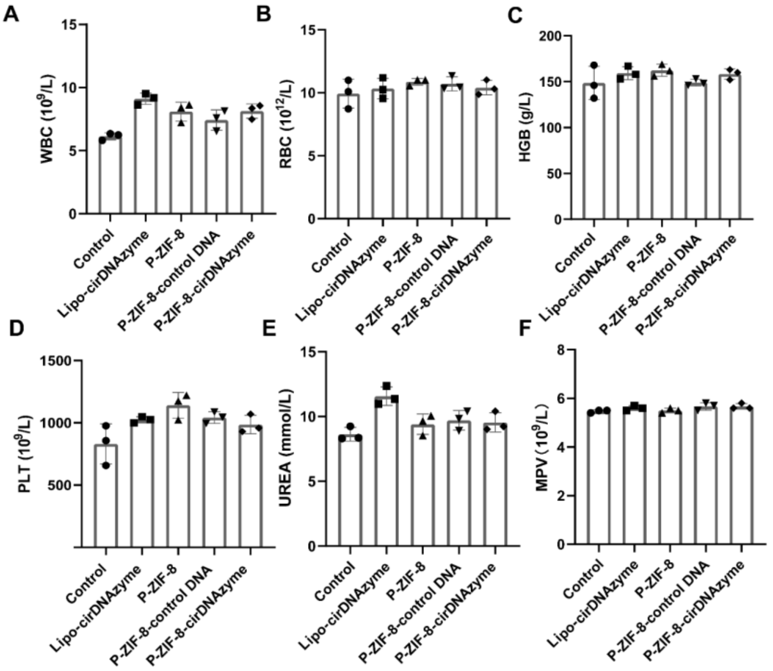

Figure S13. Blood biochemical levels and haematological parameters of the mice after treatment with different groups. All data are shown as the mean ± SD, n=3. Serum biochemistry analysis was utilized for exploring the potential systematic toxicity of different formulations. All these key parameters of the nanoparticle-treated mice were in good accordance with that of healthy mice, indicating minimal systematic toxicity of the proposed nanoparticles.

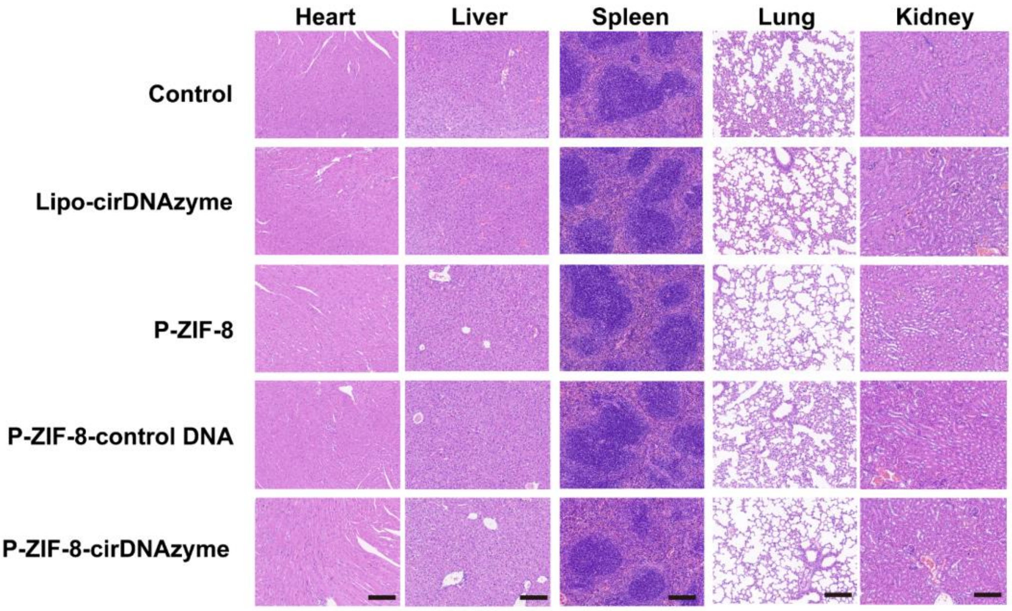

Figure S14. H&E staining of major organs separated from different groups. To investigate the potential toxicity of different formulations on major organs, the differently treated mice were sacrificed, and the collected heart, liver, spleen, lungs,

and kidneys were analyzed by using hematoxylin and eosin (H&E) staining. No physiological abnormality was visualized in these organs, verifying the minimal systematic toxicity of these nanoparticles. Scale bar: 200 μm.

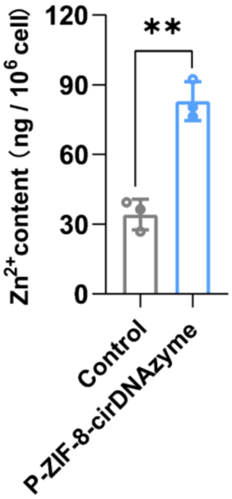

Figure S15. ICP-MS assay the change of Zn^{2+} levels after P-ZIF-8-cirDNAyzme treatment in liver aHSC. Compared with PBS (control) group, after P-ZIF-8-cirDNAyzme treatment, the Zn^{2+} content in aHSC increased, which proved P-ZIF-8-cirDNAyzme could target aHSC and induce Zn^{2+} level change in aHSC. The data are shown as the mean ± SD, n=3. Statistical analysis was performed using two-tailed unpaired Student's t-tests. $^{**}P < 0.01$.

Table S1. MMGBSA analysis of ΔG of P4H and substrate peptide under Fe^{2+} and Zn^{2+} system.

Energy component	Fe^{2+}/(kcal/mol)	Zn^{2+}/(kcal/mol)
ΔE_{VDW}	-47.022±2.422	-43.896±3.10
ΔE_{ELE}	-16.228±2.650	-15.854±2.624
ΔE_{GB}	43.570±7.193	45.056±8.508
ΔE_{SURF}	-6.440±0.211	-4.910±0.339
ΔG_{GAS}	-63.250±9.027	-59.750±9.809
ΔG_{solv}	37.130±7.179	40.145±8.454
ΔG_{bind}	-26.120±3.216	-19.605±3.856

a) ΔE_{VDW}: van der Waals energy values change, the contact area and amino acids and hydrophobic properties; b) ΔE_{ELE}: electrostatic interaction energy values change, and charge properties of amino acids; c) ΔE_{GB}: the polar solvation free energy,

which is related to the polarity of the amino acid, is the amount of energy lost in the de-solvation process; d) ΔE_{SURF}: nonpolar solvation free energy, and polarity/nonpolar amino acid, de-solvation process loss of energy; e) ΔG_{GAS}: free energy of the combination of GAS, without considering the solvent at the time of the binding energy, and bond Angle, the dihedral Angle, etc., and see the formula; f) ΔG_{solv}: the solvation free energy, the total de-solvation energy, is usually positive, indicating a loss of de-solvation energy; g) ΔG_{bind}: total binding free energy, affinity, intuitive reaction. The binding energy was calculated according to the formula using the gmx-MMPBSA program from the kinetic simulation trajectory file.

Table S2. *TIMP-1* cirDNAzyme and substrate

Name	Sequence (5'~3')
DNAzyme	CGTAAATCAGTCACCAGCATGGGGCACAGT<br>CCGAGCCGGTCGAAACAGGCCTTATGCTGG
DNAzyme'	TGACTGATTTACGCCAGCAACGGGCACAGT<br>CCGAGCCGGTCGAAACAGGCCTTATGCTGG
control DNA	CGTAAATCAGTCACCAGCATGGGGCACAGT<br>CCCCCCCCCCCCCCACAGGCCTTATGCTGG
TIMP-1 substrate	GTTCCAGTAAGGCCTGT/rA/GCTGTGCCCCA<br>CCCCACCCACAGACAG

a) the red sequence indicates the catalytic domain that can be specifically activated with Zn^{2+}. b) the orange sequence indicates the catalytic domain that is changed without catalytic activity. c) rA in *TIMP-1* substrate represents ribonucleic adenine, which is more liable to cleavage than deoxyribonucleotides.

Table S3. Primers used for RT-qPCR.

Name	Sequence (5'~3')
TIMP-1 Forward primer	AGACACGCTAGAGCAGATACC
TIMP-1 Reverse primer	GGCACAGCTACAGGCTTTAC
β-actin Forward primer	GGAGATTACTGCCCTGGCTCCTA
β-actin Reverse primer	GACTCATCGTACTCCTGCTTGCTG
